# TGF-β-Induced Endothelial to Mesenchymal Transition Is Determined by a Balance Between SNAIL and ID Factors

**DOI:** 10.3389/fcell.2021.616610

**Published:** 2021-02-12

**Authors:** Jin Ma, Gerard van der Zon, Manuel A. F. V. Gonçalves, Maarten van Dinther, Midory Thorikay, Gonzalo Sanchez-Duffhues, Peter ten Dijke

**Affiliations:** ^1^Department of Cell Chemical Biology, Leiden University Medical Center, Leiden, Netherlands; ^2^Oncode Institute, Leiden University Medical Center, Leiden, Netherlands

**Keywords:** bone morphogenetic protein, endothelial cell, EndMT, inhibitor of DNA binding, transcription factor, transforming growth factor-β2

## Abstract

Endothelial-to-mesenchymal transition (EndMT) plays an important role in embryonic development and disease progression. Yet, how different members of the transforming growth factor-β (TGF-β) family regulate EndMT is not well understood. In the current study, we report that TGF-β2, but not bone morphogenetic protein (BMP)9, triggers EndMT in murine endothelial MS-1 and 2H11 cells. TGF-β2 strongly upregulates the transcription factor SNAIL, and the depletion of *Snail* is sufficient to abrogate TGF-β2-triggered mesenchymal-like cell morphology acquisition and EndMT-related molecular changes. Although SLUG is not regulated by TGF-β2, knocking out *Slug* also partly inhibits TGF-β2-induced EndMT in 2H11 cells. Interestingly, in addition to SNAIL and SLUG, BMP9 stimulates inhibitor of DNA binding (ID) proteins. The suppression of *Id1*, *Id2*, or *Id3* expression facilitated BMP9 in inducing EndMT and, in contrast, ectopic expression of ID1, ID2, or ID3 abrogated TGF-β2-mediated EndMT. Altogether, our results show that SNAIL is critical and indispensable for TGF-β2-mediated EndMT. Although SLUG is also involved in the EndMT process, it plays less of a crucial role in it. In contrast, ID proteins are essential for maintaining endothelial traits and repressing the function of SNAIL and SLUG during the EndMT process. These data suggest that the control over endothelial vs. mesenchymal cell states is determined, at least in part, by a balance between the expression of SNAIL/SLUG and ID proteins.

## Introduction

In diverse physiological and pathological processes, endothelial cells show remarkable plasticity as they lose endothelial properties and differentiate into mesenchymal cells, a process termed endothelial-to-mesenchymal transition (EndMT) (Dejana and Lampugnani, 2018). EndMT is a gradual and reversible dynamic process, which shares similarities with epithelial-to-mesenchymal transition (EMT) (Saito, 2013). Epithelial cells can acquire different EMT features with mixed epithelial/mesenchymal phenotype. This has recently been described as epithelial cell plasticity (Yang et al., 2020). Unlike EMT, however, our current understanding of the molecular mechanisms that control EndMT are limited. When cobblestone-shaped endothelial cells (ECs) undergo EndMT, they gradually lose their tight junctions and acquire a mesenchymal-like identity and the appearance of elongated fibroblasts. In order to monitor the transition of ECs toward fibroblast-like cells, a number of proteins can be studied. For example, during EndMT the cells progressively express less cell-cell adhesion- and endothelial-specific proteins, such as vascular endothelial (VE)-cadherin, platelet/EC adhesion molecule-1 (CD31/PECAM-1), tyrosine kinase with immunoglobulin-like, and epidermal growth factor (EGF)-like domains 1 (TIE1), TIE2, and von Willebrand factor (vWF), while mesenchymal factors accumulate, including N-cadherin, α-smooth muscle actin (α-SMA), smooth muscle protein 22α (SM22α), fibronectin, and fibroblast specific protein-1 (FSP-1). EndMT participates in different physiological and pathological processes (Potenta et al., 2008; Yoshimatsu and Watabe, 2011; Hong et al., 2018; Souilhol et al., 2018). Thus, specific modulation of EndMT may provide a therapeutic benefit. For example, targeting of EndMT might be beneficial in treating human disorders, whereas controlled induction of EndMT might be a potential application in tissue engineering and wound healing (Ma et al., 2020). To allow for clinical translation of EndMT modulating approaches, more insights into the underlying molecular and cellular mechanisms of EndMT are needed.

As a complex biological process, EndMT is triggered by, among other factors, numerous cytokines and is modulated by diverse signaling pathways. Extensive studies have shown that TGF-β family proteins, including TGF-βs (Van Meeteren and Ten Dijke, 2012), activins and bone morphogenetic proteins (BMPs) (Zeisberg et al., 2007; Medici et al., 2010; Zhang et al., 2018), all have a pivotal function in controlling EndMT, as well as, EMT (Miyazono, 2009). TGF-β2 was found to be the most potent TGF-β isoform in inducing EndMT, and the most relevant TGF-β isoform involved in regulating EndMT during heart cushion development (Camenisch et al., 2002b; Azhar et al., 2009). However, knowledge regarding the effects and mechanisms by which BMPs control EndMT, remains incomplete. For instance, BMP4 activates EndMT, whereas its close relative BMP7 has been reported to antagonize it (Zeisberg et al., 2007; Medici et al., 2010). Notably, the effect of BMP9 on EndMT remains to be determined despite the fact that BMP9 is one of the major BMP ligands in ECs (Suzuki et al., 2010; Dyer et al., 2014).

TGF-β and BMPs initiate cellular responses by binding to specific cell surface transmembrane type I and type II receptors, including type I (TβRI) and type II (TβRII) receptors for TGF-β; and BMP type I receptors (BMPRIs) and BMP type II receptor (BMPRII) for BMPs (Massagué, 2008). Upon ligand-induced heteromeric complex formation, the activated receptor complex phosphorylates receptor-regulated downstream effectors, termed SMADs. TβRI induces phosphorylation of SMAD2/3 and BMPRI mediates phosphorylation of SMAD1/5/8 (Derynck and Budi, 2019). Activated R-SMADs partner with the common mediator SMAD4 and translocate into the nucleus, where they induce the expression of specific sets of target genes, such as *plasminogen activator inhibitor-1* (*PAI1*) and *Ids* (*Id1, Id2, Id3*, and *Id4*). The former and latter genes are upregulated by exposure to TGF-β and BMPs stimuli, respectively (Pepper et al., 1991; Dennler et al., 1998; Hollnagel et al., 1999; Korchynskyi and Ten Dijke, 2002).

The members of the SNAIL family of transcription factors, which includes SNAIL and SLUG (a.k.a. SNAIL1 and SNAIL2, respectively), contain Cys_2_His_2_ zinc-finger motifs that bind to E-boxes (5′-CAGGTG-3′) located in certain gene promoters (Nieto, 2002). Upon TGF–β and BMP stimulation, SNAIL proteins are upregulated and bind to the promoters of endothelial–related genes to decrease endothelial protein expression, resulting in ECs with diminished endothelial function and gradually enhanced acquisition of a mesenchymal cell morphology. These EMT–inducing transcription factors may work together and have specific functions in orchestrating the complex EndMT/EMT process (Yang et al., 2020). Of notice, there are somewhat conflicting reports regarding the role of ID proteins in EMT and EndMT, in part likely caused by cellular context–dependent factors. Indeed, although high ID1 expression has been correlated with enhanced EMT in advanced bladder tumor stages (Hu et al., 2013), and promotion of carcinogenesis and metastasis in lung cancer (Castañón et al., 2017), ID1 has also been described to antagonize EMT in mouse NMuMG mammary epithelial cells (Kondo et al., 2004). ID proteins have been shown to dimerize with the transcription factor E2A, which promotes EMT by directly binding to gene regulatory sequences (Massari and Murre, 2000). Thus, how TGF–β and BMPs regulate EndMT and how does the interplay between SNAIL family members and ID proteins influences EndMT, remain poorly understood.

In the present study, we investigated whether the TGF–β family ligands TGF–β2 and BMP9 are capable of inducing EndMT in two murine endothelial cell lines, i.e., pancreatic microvascular endothelial cells (MS–1) and lymphatic endothelial cells (2H11). We show that TGF–β2 upregulates specific EndMT transcription factors and that SNAIL, in particular, is required for EndMT. In contrast, BMP9 fails to induce full–fledged EndMT, although it strongly induces transient expression of EndMT–associated transcription factors. Mechanistically, IDs that accumulate in response to BMP9 stimulation, exhibit opposite effects to those triggered by SNAIL family proteins and antagonize TGF–β2–mediated EndMT. Given the role of TGF–β and BMPs in regulating EndMT in postnatal disease processes, our results provide important insights that may guide future therapeutic interventions based on the modulation of EndMT.

## Materials and Methods

### Materials

Recombinant human TGF–β2 was a kind gift from Joachim Nickel, University of Wurzburg. Human BMP9 (3209–BP/CF) and mouse BMP9 (5566–BP) were obtained from R&D systems. Human BMP6 was a kind gift from Slobodan Vukicevic, University of Zagreb. Human TGF–β1 (HZ–1011) was purchased from HumanZyme. Human TGF–β3 was a kind gift from Andrew Hinck, University of Pittsburgh. Mouse TGF–β2 (7346–B2) was purchased from Bio–Techne. Puromycin (P9620) was obtained from Sigma–Aldrich. The Blasticidin (R21001) and the ID proteins chemical inhibitor AGX51 (HY–129241), were purchased from Invitrogen to MedChem Express, respectively.

### Cell Culture

Murine pancreatic microvascular endothelial cells (MS–1) and murine lymphoid endothelial cells (2H11) were cultured on 0.1% (w/v) gelatin (G1890, Sigma–Aldrich) in Dulbecco’s modified Eagles’s medium (DMEM, 11965092, Thermo Fisher Scientific) supplemented with 10% fetal bovine serum (FBS, 16000044, Thermo Fisher Scientific) and 100 IU ml^–1^ penicillin/streptomycin. Human embryonic kidney 293T cells were cultured in DMEM supplemented with 10% FBS and 100 IU ml^–1^ penicillin/streptomycin. All cell lines were maintained in a 5% CO_2_ humidified air incubator at 37°C and were regularly checked for the absence of mycoplasma infection.

### Quantitative Reverse Transcription PCR (RT-qPCR)

Total RNAs were isolated using the NucleoSpin RNA II kit (740955, BIOKE) according to the instructions provided by the manufacturer. After quantifying the RNA concentration by Nanodrop (Isogen, Maarssen, Netherlands), reverse transcription was performed on the same amount of RNA using the RevertAid First Strand cDNA Synthesis Kit (K1621, Thermo Fisher Scientific). After the cDNA synthesis, RT-qPCR was conducted with GoTaq qPCR Master Mix (A6001, Promega) using the CFX Connect Detection System (1855201, Bio-Rad). The expression levels of all target genes were determined using the ΔΔCt method and were normalized for *Gapdh* expression on a per sample basis. All DNA primer sequences that were used in the study are shown in Supplementary Table S1.

### Western Blot Analysis

Cells were lysed in radioimmunoprecipitation assay (RIPA) lysis buffer containing a protease inhibitor cocktail (11836153001, Roche). After spinning down cellular debris at 1.2 × 10^4^ × g for 5 min, protein concentrations were quantified by using the bicinchoninic acid (BCA) protein assay kit (23235, Thermo Fisher Scientific). Next, the proteins were boiled for 5 min and then loaded and separated through sodium dodecyl sulfate polyacrylamide gel electrophoresis (SDS-PAGE). The resolved proteins were then transferred onto 45 μm polyvinylidene difluoride (PVDF) membranes (IPVH00010, Merck Millipore). After blocking the membranes with 5% non-fat dry milk for 1 h at room temperature in tris-buffered saline with Tween 20 (TBST), the membranes were sequentially incubated with primary and secondary antibodies. The signals were visualized by using the Clarity^TM^ Western ECL Substrate (1705060, Bio-Rad) and the ChemiDoc Imaging System (17001402, Bio-Rad). The primary antibodies used for immunoblotting were diluted 1,000 fold in TBST and were raised against the following proteins: phospho-SMAD1/5 (9516S, Cell Signaling), phospho-SMAD2 (pSMAD2, home-made (Persson et al., 1998), SNAIL (3879, Cell Signaling), SLUG (9585, Cell Signaling), SMAD1 (6944S, Cell Signaling), SMAD2 (3103S, Cell Signaling), ID1 (sc-133104, Santa Cruz), ID2 (sc-489, Santa Cruz), ID3 (sc-490, Santa Cruz), α/β-Tubulin (2148, Cell Signaling), glyceraldehyde 3-phosphate dehydrogenase (GAPDH, MAB374, Merck Millipore), Vinculin (V9131, Sigma-Aldrich). Western blotting for GAPDH, Tubulin or Vinculin were performed to serve as protein loading controls. All experiments were repeated at least three times, and representative results are shown. Use of technical or biological replicates is indicated in the figure legends. Protein amounts were quantified by densitometry using ImageJ (National Institutes of Health, United States).

### Lentiviral Vector Production and Stable Transduction of Cell Lines

Lentiviral vectors were produced in HEK293T cells by co-transfecting each of the lentiviral vector transfer constructs together with expression plasmids pCMV-VSVG, pMDLg-RRE (gag/pol), and pRSV-REV using polyethyleneimine (PEI) as previously described (Zhang et al., 2012). The transfection medium was replaced by fresh medium after 24 h and, after 48 h, harvested cell supernatants were centrifuged at 200 × g for 3 min and filtered through 0.45 μm pore-sized filters (4614, Pall Corporation). The clarified supernatants containing the lentiviral vector particles were stored at −80°C until further use. To generate *Snail* and *Slug* knockout cell lines, 2H11 and MS-1 cells were firstly transduced by adding Cas9-expressing lentiviral vector (Cas9BST-1EA, Sigma-Aldrich) supernatant together with 10 μg ml^–1^ of polybrene (107689, Sigma-Aldrich) for 24 h. The transduced cells were selected with 4 μg ml^–1^ blasticidin for 48 h to obtain stable Cas9-expressing cells. Next, the cells were transduced with lentiviral vectors expressing guide RNAs targeting *Snail* or *Slug* and were further selected by adding puromycin (1 μg ml^–1^ for MS-1 and 4 μg ml^–1^ for 2H11). The depletion of SNAIL and SLUG protein efficiencies were determined by western blot analysis.

The annotated map and sequence of the gRNA acceptor lentiviral vector construct AA19_pLKO.1-puro.U6.sgRNA.*Bve*I-Dys.Stuffer are available in Supplementary Figure S1. This plasmid was generated by inserting into the *Bcl*I site of pLKO.1-puro.U6.sgRNA.*Bfu*AI.stuffer (Addgene plasmid #50920) a 3431-bp DNA fragment derived from the human dystrophin-coding sequence (Kearns et al., 2014). This stuffer DNA segment contains four additional *Bve*I recognition sites required for efficient *Bve*I plasmid digestion. The digestion of AA19_pLKO.1-puro.U6.sgRNA.*Bve*I-Dys.Stuffer with *Bve*I creates CGGT and GTTT 5′ overhangs permitting directional ligation to the ACCG and AAAC 5′ overhangs, respectively, of annealed oligodeoxyribonucleotides corresponding to a gRNA spacer (Maggio et al., 2016). Spacer sequences of *Snail*– and *Slug*–targeting gRNAs were identified by running the CHOPCHOP algorithm http://chopchop.cbu.uib.no/ (Montague et al., 2014; Labun et al., 2019). The lists of candidate gene knockout gRNAs were shortened by additional screening for potential off–target activity with the aid of Cas–OFFinder http://www.rgenome.net/cas-offinder/ (Bae et al., 2014). The oligonucleotide sequences corresponding to the selected gRNAs are listed in Supplementary Table S2. Lentiviral vectors for the expression of mouse *Id1*, *Id2*, and *Id3* were made and used to generate stable cell lines overexpressing these proteins. In brief, the pLV–*Id1* plasmid was made by digesting pcDNA3–*Id1* with *Nde*I (ER0581, Thermo Fisher Scientific) and *Xho*I (ER0692, Thermo Fisher Scientific) and isolating and subcloning the *Id1* fragment into pLV–IRES–Puro containing a FLAG–tag at the N–terminal cut with same two enzymes. The pLV–*Id2* and pLV–*Id3* constructs were made by cloning the *Id2* and *Id3* fragments isolated from *Bcu*I (ER1251, Thermo Fisher Scientific) and *Xba*I (ER0682, Thermo Fisher Scientific) digested pCDEF3–Id2 or pBluescript KS(–)–*Id3* vectors into the same enzymes cut pLV–IRES–Puro with a FLAG–tag at the N–terminal, respectively. To generate MS–1 and 2H11 stable cell lines overexpressing ID1, ID2, or ID3, the respective cells were exposed for 24 h to clarified supernatants containing lentiviral vectors expressing *Id1*, *Id2*, or *Id3* and polybrene at a final concentration of 10 μg ml^–1^. At 48 h post-transduction, the cells were subjected to puromycin selection (1 μg ml^–1^ for MS-1 and 4 μg ml^–1^ for 2H11). The ectopic expression of ID1, ID2, and ID3 was checked at both the RNA and protein levels.

### Cell Proliferation Assay

One thousand MS-1 and 2H11 cells in 200 μl of regular culture medium were seeded in wells of a 96-well microplate Essen ImageLock^TM^ (4379, Essen Bioscience). Thereafter, the plate was placed in an IncuCyte ZOOM instrument (Essen Bioscience). The cells were monitored in real-time by taking images every 4 h for 2 days in total. The relative cell confluence was analyzed and quantified through the instrument’s software.

### *In vitro* Migration Assay

Approximately 2.5 × 10^4^ MS-1 and 2H11 cells in 100 μl of regular culture medium were seeded in wells of a 96-well microplate Essen ImageLock^TM^ and were incubated overnight to allow for cell attachment. Subsequently, a WoundMaker^TM^ device (4563, Essen Bioscience) containing 96 pins was used to scratch homogeneous micron-wide wounds through the cell monolayers. After the removal of debris and detached cells, the monolayers were washed with serum-free medium twice, after which 100 μl of fresh serum-free medium was added to each well. Then the plate was placed in the IncuCyte instrument, and the cells were monitored in real-time by acquiring images every 2 h for 22 h with the wound width being analyzed by the instrument’s software.

### Small Interfering RNA Transfections

MS-1 cells were transfected with 40 nM of non-targeting (NT; 4390843, Dharmacon), *Id1* (*Id1*; L-040701-01-0005, Dharmacon), *Id2* (*Id2*; L-060495-00-0005, Dharmacon), or *Id3* (*Id3*; L-046495-00-0005, Dharmacon) small interfering RNAs (siRNAs) mixed with siRNA transfection reagent DharmaFECT 1 (T-2001, GE Healthcare Dharmacon). 2H11 cells were transfected with 80 nM of NT or *Id1/2/3* siRNAs using DharmaFECT 3 transfect reagent (T-2003, GE Healthcare Dharmacon). The siRNA and transfection reagent mixtures were incubated in serum-free medium for 20 min at room temperature before being added to the cells. The cells subjected to target gene knockdown were analyzed at 24 h post-transfection. The target gene knockdown frequencies were assessed by mRNA expression analyses using qRT-PCR.

### EndMT Assays

MS-1 and 2H11 cells were cultured in wells of 6-well plates in medium containing 10% FBS and were subsequently treated with recombinant human TGF-β2 (1 ng ml^–1^) or recombinant human BMP9 (5 ng ml^–1^) for 3 days to investigate their effects on EndMT. Where indicated, lower TGF-β2 concentrations were also used to induce EndMT. For example, MS-1 and 2H11 cells were treated for 3 days with TGF-β2 at 0.1 and 0.2 ng ml^–1^, respectively, and cell morphology changes were assessed. The cell morphology was quantified by measuring the cell elongation ratios. This was performed by calculating the ratio of cell length to cell width using ImageJ. Cell elongation ratios of fifty cells in each experiment were determined and results from three independent biological replicates are presented.

### Immunofluorescence Staining

After stimulating the cells with TGF-β2 or BMP9, the cells were fixed with 4% formalin and were permeabilized with 0.1% triton X-100. Subsequently, the MS-1 cell samples were blocked with 3% bovine serum albumin (BSA, A-6003, Sigma-Aldrich) in phosphate-buffered saline (PBS) for 45 min at room temperature and then incubated with primary antibodies directed against PECAM-1 (1:500, 553370, Becton Dickinson) and SM22α (1:500, ab14106, Abcam); whereas 2H11 samples were incubated with a primary antibody raised against SM22α (1:500, ab14106, Abcam). Primary antibody incubations took place at room temperature for 45 min. After washing three times with PBS, the MS-1 cell samples were exposed to PBS containing secondary antibody donkey anti-rat Alexa 488 (1:1,000, A21208, Invitrogen) and goat anti-rabbit Alexa 594 (1:1000, A11012, Invitrogen); whereas the 2H11 cell samples were incubated with Alexa Fluor 488 Phalloidin (1:1,000, A12379, Thermo Fisher Scientific) and Goat anti-rabbit Alexa 594 at a dilution of 1:1,000. Secondary antibody treatments took place at room temperature in the dark for 45 min. The nuclei were stained with 4′,6-diamidino-2 phenylindole (DAPI, H-1200, Vector Laboratories). Images were taken by confocal microscopy (SP8, Leica Microsystems). The intensity of the fluorescence signals in each confocal image was quantified by ImageJ. All experiments were repeated at least three times, and representative results are shown.

### Statistical Analyses

Results were compared by unpaired Student’s *t*–test. Differences were considered significant when *p* < 0.05.

## Results

### TGF–β2 Induces EndMT Whilst BMP9 Does Not

TGF–β family proteins are key regulators of endothelial cell function (Ma et al., 2020) and TGF–β ligands are known as the most important drivers of EndMT (Suzuki et al., 2010; Ten Dijke et al., 2012). Among the three TGF–β isoforms (i.e., TGF–β1, TGF–β2, and TGF–β3), TGF–β2 has been linked as a pivotal factor in EndMT during atrioventricular (AV) cushion formation (Camenisch et al., 2002a; Sabbineni et al., 2018), being therefore chosen as primary TGF–β isoform in our experimental studies. The TGF–β family member BMP9 has been shown to mediate vascular quiescence and stimulate proliferation of stem cell–derived endothelial cells (MESECs), by activating high–affinity receptors in these cells (Suzuki et al., 2010). In order to gain deeper insights into the mechanisms by which these two cytokines regulate endothelial (dys)function, we characterized the EndMT response in the murine endothelial cell lines MS–1 and 2H11 cells for which previously TGF–β was found to induce a prominent EndMT response (Walter–Yohrling et al., 2004; Mihira et al., 2012). We first investigated the expression of TGF–β receptors by RT–qPCR in these two cell lines. As shown in Supplementary Figures S2A,B, both cell lines express ALK5 (encoded by *Tgfbr1*), ALK1 (encoded by *Acvrl1*), TGFβRII (encoded by *Tgfbr2*), BMPRII (encoded by *Bmpr2*), betaglycan (encoded by *Tgfbr3*) (albeit at relatively low levels), and endoglin (encoded by *Eng*) mRNA, suggesting that they are signaling proficient for TGF–β and BMP9.

**FIGURE 1:**
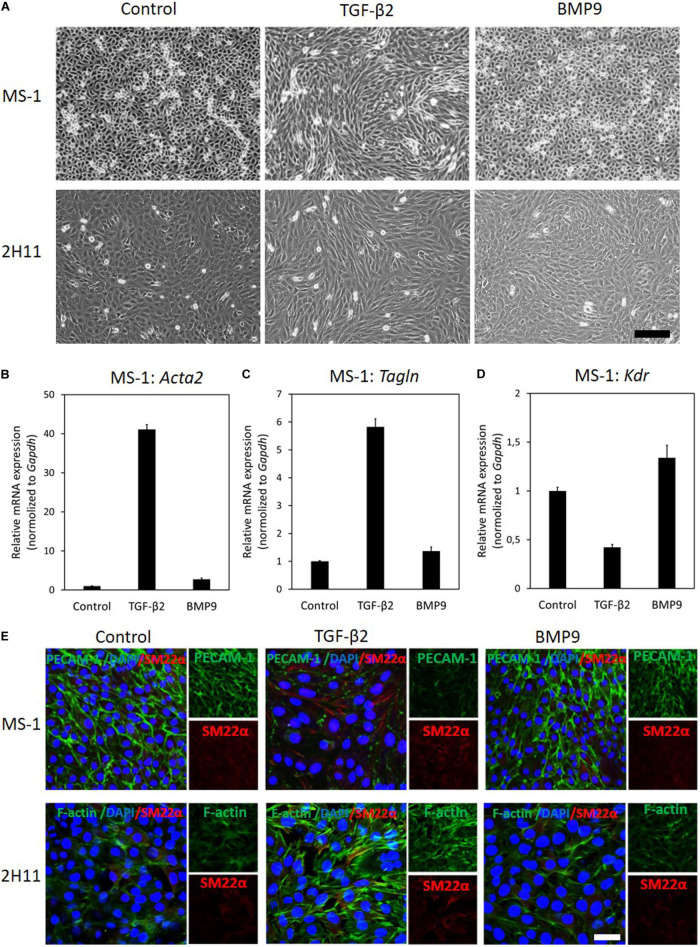
TGF–β2 induces EndMT whilst BMP9 does not. **(A)** Assessing cell morphological changes induced by TGF–β2 and BMP9. Brightfield microscopy images of MS–1 (upper panel) and 2H11 (lower panel) cells showing distinct cell morphologies (i.e., cobblestone or fibroblast–like) after TGF–β2 (1 ng ml^–1^) and BMP9 (5 ng ml^–1^) treatments for 3 days. Scale bar: 200 μm. **(B–D)** RT-qPCR analysis of endothelial and mesenchymal markers in MS-1 cells. MS-1 cells were exposed to medium containing TGF-β2 (1 ng ml^–1^) or BMP9 (5 ng ml^–1^) or medium containing ligand buffer (control) for 3 days. The expression of mesenchymal cell marker genes *Acta2* **(B)** and *Tagln* **(C)** and endothelial cell marker gene *Kdr* **(D)** was quantified by RT-qPCR. Expression levels were normalized to those of the housekeeping gene *Gapdh*. Results are expressed as mean ± SD. Representative results from three independent experiments are shown. **(E)** Fluorescence microscopy analysis of endothelial and mesenchymal markers in MS-1 and 2H11 cells. MS-1 and 2H11 cells were incubated in medium containing TGF-β2 (1 ng ml^–1^) or BMP9 (5 ng ml^–1^) or medium containing ligand buffer (control) for 3 days. The expression of the endothelial cell marker PECAM-1 (green) and mesenchymal cell marker SM22α (red) in nuclei (blue) stained MS-1 cells (upper panel) and mesenchymal cell markers F-actin (green) and SM22α (red) in nuclei (blue) stained 2H11 cells (lower panel) were assessed by using immunofluorescent staining, respectively. Representative results from at least three independent experiments are shown. Scale bar: 50 μm.

Next, MS–1 and 2H11 cells were treated with TGF–β2 or BMP9 for 3 days to study whether they have an effect on EndMT. We started by examining the effects of these treatments on MS–1 cell morphology. In the absence of exogenous ligand stimulation, MS–1 cells displayed a cobblestone–like phenotype and tended to remain closely attached to each other. However, upon challenge with TGF–β2 for 3 days, the MS–1 cells showed a spindle–shaped morphology (Figure 1A, upper panel). Interestingly, upon treatment with BMP9 for 3 days, no apparent morphological changes were observed (Figure 1A, upper panel). To confirm this change in cell shape, the elongation ratios of individual cells were measured and plotted in Supplementary Figure S3A. Moreover, to determine whether this lack of effect is specific for BMP9, we challenged the MS–1 cells with BMP6. After addition of BMP6, which, in contrast to BMP9, signals via ALK2 instead of ALK1, also no loss of endothelial morphology was observed in MS–1 cells (Supplementary Figure S4). This data suggests that MS–1 cells undergo EndMT in response to TGF–β2, whilst BMP9 does not seem capable of inducing EndMT, at least in a robust fashion.

Next, we sought to confirm these findings by studying the expression of EndMT-related markers. As shown in Figures 1B,C, the mRNA expression levels of the mesenchymal cell markers genes *Acta2* (encoding α-SMA) and *Tagln* (encoding SM22α) were significantly increased in the presence of TGF-β2, yet they were only slightly altered by BMP9 exposure. Besides, the expression of the endothelial marker *Kdr* (encoding vascular endothelial growth factor receptor) was attenuated by TGF-β2, while it was promoted by BMP9 (Figure 1D). These results indicate a possible role of BMP9 in maintaining endothelial properties. Consistent with these findings, microscopy analysis of EndMT-related protein expression by immunofluorescence staining revealed that the synthesis of the endothelial protein PECAM-1 was strongly down-regulated in response to TGF-β2, yet it was barely affected upon BMP9 treatment (Figure 1E). The expression of the mesenchymal protein SM22α was profoundly enhanced by TGF-β2, while no effect was observed on its expression upon BMP9 stimulation. The quantification of the PECAM-1 and SM22α fluorescence intensity in MS-1 is shown in Supplementary Figures S5A,B. Thus, we conclude that, in contrast to BMP9, TGF-β2 induces robust EndMT in MS-1 cells.

To further investigate EndMT responses of ECs to TGF-β2 and BMP9, we performed similar experiments in 2H11 cells. In response to TGF-β2 treatments for 3 days, 2H11 cells became elongated and acquired a mesenchymal-like morphology, which was not observed when these cells were exposed, also for 3 days, to BMP9 (Figure 1A, lower panel). To confirm this change in cell shape, the elongation ratios of individual cells were measured and plotted in Supplementary Figure S3B. Next, the expression of the EndMT marker SM22α and that of the filamentous actin (F-actin) stress fibers were checked by microscopy analysis using immunodetection and fluorophore-conjugated phalloidin staining, respectively. As shown in Figure 1E (lower panel), TGF-β2 augmented SM22α and F-actin amounts, while BMP9 did not. The quantification of the F-actin and SM22α fluorescence intensity in 2H11 is shown in Supplementary Figures S5C,D. These results demonstrate that, similarly to MS-1 cells, 2H11 cells respond to TGF-β2 by undergoing EndMT, whereas they are incapable of doing so once treated with BMP9.

### The Effects of TGF-β2 and BMP9 on SNAIL and SLUG Expression in ECs

**FIGURE 2:**
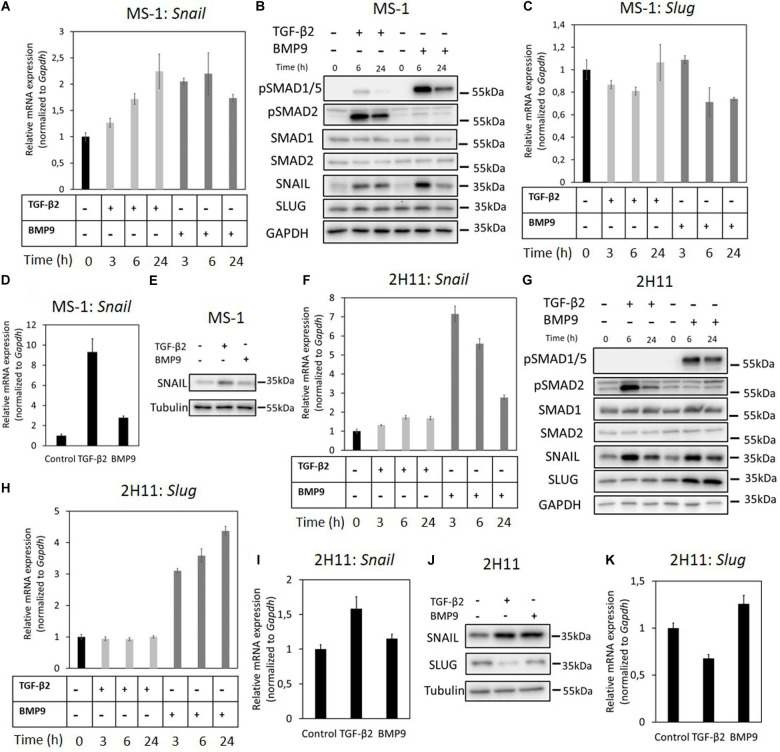
Effects of TGF-β2 and BMP9 on SNAIL and SLUG expression. **(A)** RT-qPCR analysis of the effects of TGF-β2 (1 ng ml^–1^) and BMP9 (5 ng ml^–1^) on *Snail* mRNA expression after 3, 6, and 24 h treatments in MS-1 cells. **(B)** Western blot analysis of the effects of TGF-β2 (1 ng ml^–1^) and BMP9 (5 ng ml^–1^) on SNAIL and SLUG protein expression after 6 and 24 h treatments in MS-1 cells. **(C)** RT-qPCR analysis of the effects of TGF-β2 (1 ng ml^–1^) and BMP9 (5 ng ml^–1^) on *Slug* mRNA expression after 3, 6, and 24 h treatments in MS-1 cells. **(D)** RT-qPCR analysis of the effects of TGF-β2 (1 ng ml^–1^) and BMP9 (5 ng ml^–1^) on *Snail* mRNA expression after 3 days treatments in MS-1 cells. **(E)** Western blot analysis of the effects of TGF-β2 (1 ng ml^–1^) and BMP9 (5 ng ml^–1^) on SNAIL protein expression after 3 days treatments in MS-1 cells. **(F)** RT-qPCR analysis of the effects of TGF-β2 (1 ng ml^–1^) and BMP9 (5 ng ml^–1^) on *Snail* mRNA expression after 3, 6, and 24 h treatments in 2H11 cells. **(G)** Western blot analysis of the effects of TGF-β2 (1 ng ml^–1^) and BMP9 (5 ng ml^–1^) on SNAIL and SLUG proteins expression after 6 and 24 h treatments in 2H11 cells. **(H)** RT-qPCR analysis of the effects of TGF-β2 (1 ng ml^–1^) and BMP9 (5 ng ml^–1^) on *Slug* mRNA expression after 3, 6, and 24 h treatments in 2H11 cells. **(I)** RT-qPCR analysis of the effects of TGF-β2 (1 ng ml^–1^) and BMP9 (5 ng ml^–1^) on *Snail* mRNA expression after 3 days treatments in 2H11 cells. **(J)** Western blot analysis of the effects of TGF-β2 (1 ng ml^–1^) and BMP9 (5 ng ml^–1^) on SNAIL and SLUG proteins expression after 3 days treatments in 2H11 cells. **(K)** RT-qPCR analysis of the effects of TGF-β2 (1 ng ml^–1^) and BMP9 (5 ng ml^–1^) on *Slug* mRNA expression after 3 days treatments in 2H11 cells. All the mRNA expression levels were normalized to those of the housekeeping gene *Gapdh*. Results are expressed as mean ± SD. Representative results from three independent experiments are shown.

EndMT-related transcription factors such as SNAIL and SLUG, contribute to the initiation and maintenance of EndMT processes (Kokudo et al., 2008; Cooley et al., 2014). Hence, we next aimed to study how these factors are regulated by TGF-β2 and how critical are they in inducing EndMT in MS-1 and 2H11 cells. First, *Snail* and *Slug* mRNA expression levels were examined in MS-1 cells treated with TGF-β2 or BMP9 (Figures 2A–C). After stimulation with TGF-β2, which promoted SMAD2 phosphorylation without influencing total SMAD2 protein levels, *Snail* mRNA levels were significantly increased in a time-dependent manner in MS-1 cells (Figure 2A). Indeed, *Snail* mRNA levels increased 1. 3-, 1. 7-, and 2.3-fold following 3, 6, and 24 h treatments with TGF-β2, respectively (Figure 2A). In response to BMP9, its intracellular effectors SMAD1/5 became phosphorylated in MS-1 cells (Figure 2B). Interestingly, *Snail* expression was strongly induced by BMP9, yet the maximum expression levels were not maintained with longer BMP9 incubation periods (Figure 2A). Indeed, *Snail* expression was increased 2.1- and 2.2-fold after 3 and 6 h treatments with BMP9, respectively, however, after 24 h incubations with BMP9, *Snail* expression was stimulated 1.7-fold in MS-1 cells (Figure 2A). We confirmed the TGF-β2- and BMP9-dependent induction patterns of SNAIL expression at the protein level in MS-1 cells (Figure 2B). Consistent with the changes in mRNA expression levels, increased SNAIL protein amounts were maintained in MS-1 cells treated with TGF-β2 for 6 and 24 h. In BMP9-treated MS-1 cells, these increased amounts were clearly observed at the 6 h time-point, but far less at 24 h (Figure 2B). Conversely, significant increases in SLUG expression levels were observed neither upon TGF-β2 nor BMP9 stimulation (Figures 2B,C). Quantification of the SNAIL and SLUG western blot results in MS-1 cells by densitometry analysis was performed in three independent experiments (Supplementary Figures S6A,B). Further experiments revealed that the induction of SNAIL expression by TGF-β2 and BMP9 occurred in a concentration-dependent manner, while SLUG expression was not influenced (Supplementary Figures S7A,B).

We also examined the effects of TGF-β2 and BMP9 on the expression of SNAIL in MS-1 cells exposed to these ligands for 3 days. As shown in Figure 2D, *Snail* mRNA expression levels were increased 9.3- and 2.8-fold after TGF-β2 and BMP9 treatments for 3 days, respectively. The SNAIL protein was significantly upregulated upon incubations with TGF-β2 and BMP9 for 3 days (Figure 2E). The quantification of SNAIL expression, resulting from three independent experiments, showed 2.1- and 1.6-fold increases in response to TGF-β2 and BMP9, respectively (Supplementary Figure S8A). Thus, in contrast to SLUG, SNAIL might be a key mediator of TGF-β2-induced EndMT in MS-1 cells. While BMP9 induces SNAIL, albeit in a transient “bell-shaped” response, this upregulation seems insufficient to mediate a substantial EndMT response in MS-1 cells.

Next, to further investigate the function of SNAIL and SLUG during TGF-β2-induced EndMT, we performed a similar set of experiments in the 2H11 cell line. TGF-β2 significantly enhanced the expression of SNAIL both at the mRNA and protein levels. Maximal 1.7-fold induction of *Snail* mRNA expression was reached by exposing 2H11 cells to TGF-β2 for 6 h (Figure 2F). The SNAIL protein amounts also increased after 6 and 24 h treatments with TGF-β2 (Figure 2G). BMP9 greatly stimulated *Snail* expression that reached a level 7.2-fold higher than that measured in untreated 2H11 cells at 3 h (Figure 2F). However, at the 6 and 24 h timepoints, *Snail* expression decreased to levels 5.6- and 2.8-fold higher, respectively, than those detected in BMP9-untreated 2H11 controls (Figure 2F). The synthesis of SNAIL protein was potently induced by BMP9 at 3 h, with its amounts being progressively reduced after exposing 2H11 cells to BMP9 for 6 and 24 h (Figure 2G). The quantification of these expression levels is shown in Supplementary Figure S6C. Interestingly, the expression of SLUG protein in 2H11 cells was induced 4.4-fold after a 24 h incubation period with BMP9, while it was not influenced by TGF-β2 (Figure 2G). The quantification of changes in SLUG protein expression levels is shown in Supplementary Figure S6D. In addition, *Slug* mRNA expression was strongly upregulated by BMP9, but not by TGF-β2, after 3, 6, and 24 h stimulation in 2H11 (Figure 2H). Upon a 3 days stimulation, SNAIL expression was significantly promoted by TGF-β2 and BMP9 at both the mRNA and protein levels (Figures 2I,J). In contrast, after a 3 days stimulation, *Slug* expression was unchanged by BMP9 while it was only slightly inhibited by TGF-β2 (Figure 2K). The quantification of SNAIL and SLUG expression based on three independent experiments is shown in Supplementary Figures S8B,C. This data suggests that SNAIL family factors, especially SNAIL, have a role in driving the TGF-β2-induced EndMT process in 2H11 cells.

We also investigated the effects of TGF-β2 and BMP9 on *Twist* and *Zeb1* mRNA expression. We found that *Twist* was expressed at very low levels in MS-1 cells (data not shown). In MS-1 cells, *Zeb1* was not regulated by TGF-β2, and was even suppressed by BMP9 (Supplementary Figure S9A). In contrast, in 2H11 cells, *Zeb1* was induced by TGF-β2 and was not regulated by BMP9 (Supplementary Figure S9B). In this study, we have therefore focused on *Snail* family transcription factors.

As in the previous experiments we used human ligands on mouse cell lines (i.e., MS-1 and 2H11), we next tested whether these two mouse cell lines response similarly to human and mouse TGF-β2 and BMP9. As shown in Supplementary Figures S10A–D, ligands from both species induced similar to identical responses with respect to SMAD phosphorylation and SNAIL expression. Moreover, we assessed the responses of ECs to the three different TGF-β isoforms, i.e., TGF-β1, TGF-β2, and TGF-β in MS-1 cells. As shown in Supplementary Figure S11, all three TGF-β isoforms induced SMAD2 phosphorylation and SNAIL expression in a similar manner.

Together, the results described above sparked our interest in investigating the reason as to why BMP9 greatly induces the expression of transcription factors SNAIL and SLUG and yet it cannot induce EndMT.

### Depletion of *Snail* Attenuates TGF-β2-Induced EndMT

**FIGURE 3:**
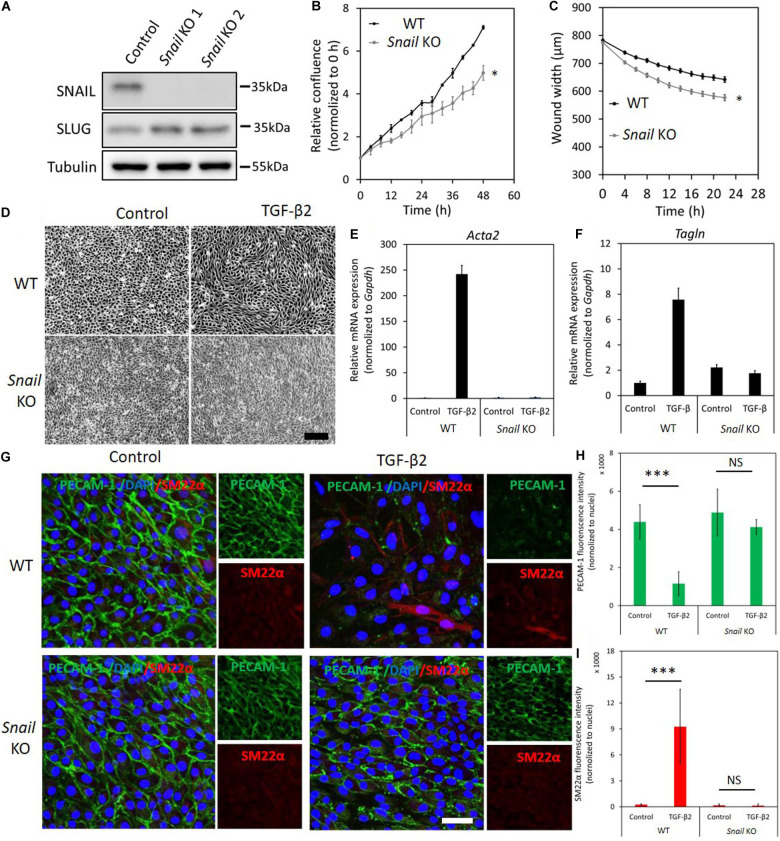
Depletion of *Snail* attenuates TGF-β2-induced EndMT in MS-1 cells. **(A)** Western blot analysis of the *Snail* depletion with two independent guide RNAs using CRISPR/Cas9-based gene editing in MS-1 cells. **(B)** The effect of *Snail* depletion on MS-1 cells proliferation. **(C)** The effect of *Snail* depletion on MS-1 cells migration ability. *p < 0.05. **(D)** Assessing cell morphological changes induced by TGF-β2 in parental MS-1 and *Snail* knockout MS-1 cells. Brightfield microscopy images of parental MS-1 (upper panel) and *Snail* knockout MS-1 (lower panel) cells showing distinct cell morphologies (i.e., cobblestone or fibroblast-like) after TGF-β2 (0.1 ng ml^–1^) treatment for 3 days. Scale bar: 200 μm. **(E,F)** RT-qPCR analysis of mesenchymal cell markers in MS-1 cells. Parental and *Snail* knockout MS-1 cells were exposed to medium containing TGF-β2 (1 ng ml^–1^) or medium containing ligand buffer (control) for 3 days. The expression of mesenchymal cell marker genes *Acta2* **(E)** and *Tagln* **(F)** was quantified by RT-qPCR. Expression levels were normalized to those of the housekeeping gene *Gapdh*. Results are expressed as mean ± SD. Representative results from three independent experiments are shown. **(G)** Fluorescence microscopy analysis of endothelial and mesenchymal cell markers in MS-1. Parental MS-1 and *Snail* knockout MS-1 cells were incubated in medium containing TGF-β2 (1 ng ml^–1^) or medium containing ligand buffer (control) for 3 days. The expression of the endothelial cell marker PECAM-1 (green) and mesenchymal cell marker SM22α (red) in nuclei (blue) stained parental MS-1 (upper panel) and *Snail* knockout MS-1 (lower panel) cells were assessed by using immunofluorescent staining, respectively. Scale bar: 50 μm. **(H,I)** Quantified mean fluorescence intensity of PECAM-1 **(H)** and SM22α **(I)**. At least six representative images from three independent experiments were quantified. Results are expressed as mean ± SD. NS, not significant; ***p < 0.001.

As the expression levels of SNAIL were greatly induced by TGF-β2 in MS-1 cells, we investigated the effect of *Snail* depletion on TGF-β2-driven/mediated EndMT by using CRISPR/Cas9-mediated gene knockouts. We generated two independent guide RNAs (gRNAs) to create insertions and deletions (INDELs) at the endogenous *Snail* gene locus resulting in the disruption of the coding sequence by frameshifting. The sequences of the oligonucleotides used for assembling the gRNA expression constructs are listed in Supplementary Table S2. The knockout of *Snail* was verified by checking the absence of SNAIL protein using western blot analysis of MS-1 cells expressing *Snail*-targeting Cas9:gRNA complexes (Figure 3A). Interestingly, SLUG expression was slightly increased upon permanent loss of SNAIL (Figure 3A). By real-time tracking of cell numbers, we found that *Snail* depletion decreased MS-1 cell proliferation and/or viability (Figure 3B). Furthermore, *Snail* knockout in MS-1 cells promoted cell migration in a wound healing assay (Figure 3C). Under routine MS-1 cell culture conditions, obvious morphological changes were observed neither in parental nor *Snail* knockout MS-1 cells (Figure 3D).

Next, we examined the effect of *Snail* depletion on TGF-β2-induced EndMT in MS-1 cells. *Snail* knockout MS-1 cells showed slightly less TGF-β2-induced morphological changes toward a mesenchymal shape when compared to parental MS-1 cells (Supplementary Figure S12). To further assess this response, we determined whether a low concentration of TGF-β2 is sufficient to induce EndMT (Supplementary Figure S13). Stimulating MS-1 cells with TGF-β2 ligand at 0.1 ng ml^–1^ for 3 days sufficed to trigger cell morphology changes. As expected, morphological changes were readily observed in parental MS-1 cells, whereas in *Snail* knockout MS-1 cells the depletion of *Snail* compromised TGF-β2-mediated EndMT morphological changes (Figure 3D), To confirm this change in cell shape, the elongation ratios of individual cells were measured and plotted in Supplementary Figure S14. This notion was strengthened by the analysis of mRNA expression levels of *Acta2* and *Tagln*, encoding the mesenchymal marker proteins α-SMA and SM22α, respectively. As shown in Figures 3E,F, the TGF-β2-dependent induction of *Acta2* and *Tagln* expression in MS-1 cells was lost upon *Snail* depletion. These results were further verified by fluorescence microscopy analysis upon immunostaining for the endothelial PECAM-1 and mesenchymal SM22α markers (Figures 3G–I). After treatment with TGF-β2 for 3 days, cultures of the parental MS-1 cells showed a significant decrease in PECAM-1 accumulation and higher frequencies of SM22α-positive cells than those detected in mock-treated controls (Figure 3G upper panel and Figures 3H,I). In contrast, in cultures of *Snail* knockout MS-1 cells, neither PECAM-1 protein amounts nor SM22α-positive cell frequencies were altered by TGF-β2 treatments (Figure 3G lower panel and Figures 3H,I). These results suggest that SNAIL is essential for TGF-β2-induced EndMT in MS-1 cells.

**FIGURE 4:**
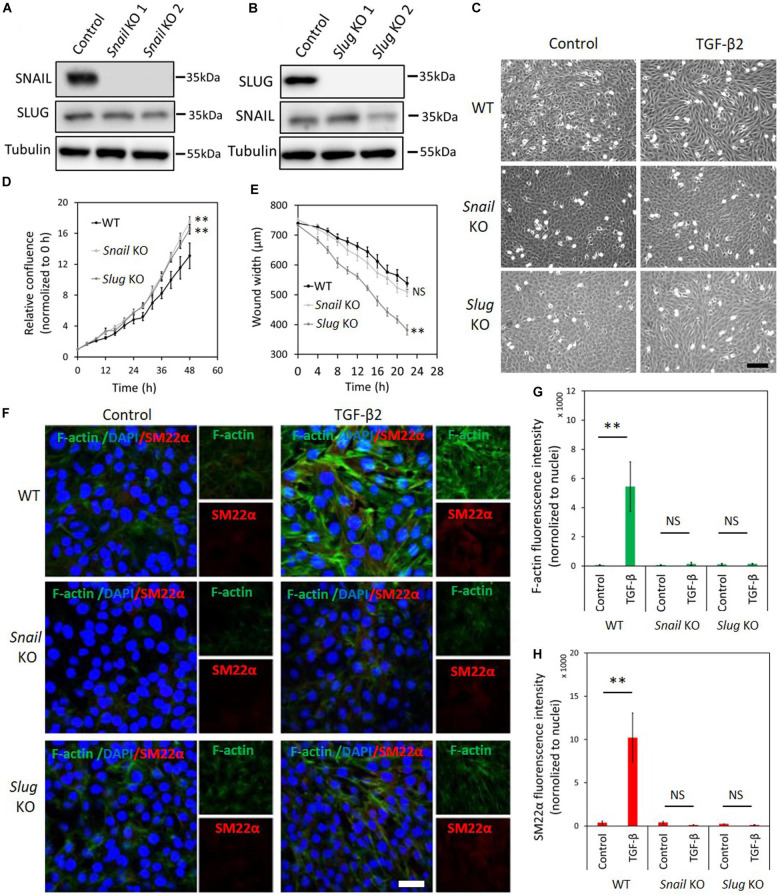
Depletion of *Snail* or *Slug* attenuates TGF-β2-induced EndMT in 2H11 cells. **(A,B)** Western blot analysis of the SNAIL **(A)** and SLUG **(B)** depletion with two independent guide RNAs using CRISPR/Cas9-based gene editing in 2H11 cells. **(C)** Assessing cell morphological changes induced by TGF-β2 in parental 2H11, *Snail* knockout 2H11 and *Slug* knockout 2H11 cells. Brightfield microscopy images of parental 2H11 (upper panel), *Snail* knockout 2H11 (middle panel) and *Slug* knockout 2H11 (lower panel) cells showing distinct cell morphologies (i.e., cobblestone or fibroblast-like) after TGF-β2 (0.2 ng ml^–1^) treatment for 3 days. Scale bar: 200 μm. **(D)** The effects of *Snail* and *Slug* depletion on 2H11 cells proliferation. **(E)** The effects of *Snail* and *Slug* depletion on 2H11 cells migration ability. **p < 0.005. **(F)** Fluorescence microscopy analysis of mesenchymal markers in 2H11 cells. Parental 2H11, *Snail* knockout and *Slug* knockout 2H11 cells were incubated in medium containing TGF-β2 (1 ng ml^–1^) or medium containing ligand buffer (control) for 3 days. The expression of mesenchymal cell markers F-actin (green) and SM22α (red) in nuclei (blue) stained parental 2H11 (upper panel), *Snail* knockout 2H11 (middle panel) and *Slug* knockout 2H11 (lower panel) cells were assessed by using immunofluorescent staining. Scale bar: 50 μm. **(G,H)** Quantified mean fluorescence intensity of F-actin **(G)** and SM22α **(H)**. At least six representative images from three independent experiments were quantified. Results are expressed as mean ± SD. NS, not significant; **p < 0.005.

To unveil the function of SNAIL and SLUG in TGF-β2-mediated EndMT in 2H11 cells, we applied two independent gRNAs to knockout either *Snail* or *Slug* by CRISPR/Cas9-mediated gene editing (Figures 4A,B). Under normal cell culture conditions, the morphology of 2H11 cells was not overtly affected upon *Snail* or *Slug* depletion (Figure 4C). Unlike in MS-1 cells, in 2H11 cells either *Snail* or *Slug* deficiencies promoted cell proliferation and/or viability (Figure 4D). Moreover, when compared with parental 2H11 cells, *Slug* knockout increased 2H11 cell migration, whereas *Snail* knockout did not (Figure 4E). Exposure to TGF-β2 failed to confer a mesenchymal morphology to *Snail* knockout 2H11 cells (Figure 4C). In contrast, *Slug* knockout cells kept responding to TGF-β2 by undergoing the morphological changes characteristic of EndMT (Figure 4C). To confirm this change in cell shape, the elongation ratios of individual cells were measured and plotted in Supplementary Figure S15. These findings were consolidated by examining the level of mesenchymal cell protein SM22α and by using Phalloidin to determine the presence and localization of F-actin fibers in 2H11 cells. As shown in Figures 4F–H, consistent with the effects on cell morphology, the loss of *Snail* dramatically blocked the TGF-β2-induced accumulation of the mesenchymal protein SM22α as well as F-actin in 2H11 cells. Interestingly, *Slug* knocked-out 2H11 cells also presented a decreased build-up of SM22α and F-actin fibers in response to TGF-β2 (Figures 4F–H). Together, our findings demonstrated that SNAIL plays a critical role in TGF-β2-mediated EndMT, while not as crucial as SNAIL, SLUG also participates to some extent in TGF-β2-mediated EndMT.

### ID Proteins Are Critical in the BMP9-Dependent Maintenance of Endothelial Cell Phenotypes

**FIGURE 5:**
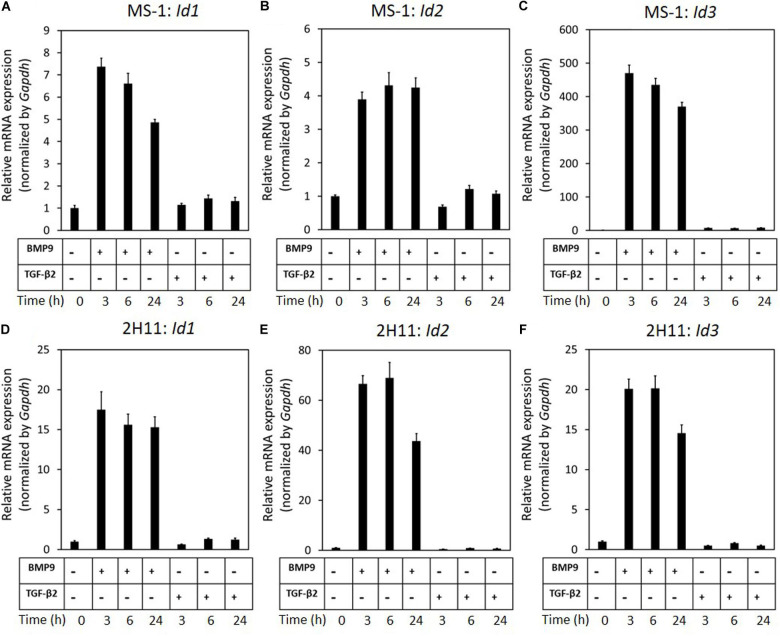
BMP9 induces *Id1*, *Id2*, and *Id3* expression, but not TGF-β2, in both MS-1 and 2H11 cells. **(A–C)** RT-qPCR analysis of the effects of BMP9 (5 ng ml^–1^) and TGF-β2 (1 ng ml^–1^) on *Id1* **(A)**, *Id2* **(B)** and *Id3* **(C)** mRNA expression after 3, 6, and 24 h treatments in MS-1 cells. **(D–F)** RT-qPCR analysis of the effects of BMP9 (5 ng ml^–1^) and TGF-β2 (1 ng ml^–1^) on *Id1* **(D)**, *Id2* **(E)**, and *Id3* **(F)** mRNA expression after 3, 6, and 24 h treatments in 2H11 cells. Expression levels were normalized to those of the housekeeping gene *Gapdh*. Results are expressed as mean ± SD. Representative results from three independent experiments are shown.

Our previous results revealed that while BMP9 strongly induces SNAIL and SLUG expression in ECs, it is unable to trigger EndMT. We hence hypothesized that BMP9, in contrast to TGF-β2, selectively induces genes that negatively regulate the EndMT process and, in these investigations, focused on BMP signaling *Id* target genes. As expected, we found that BMP9 stimulation readily induced *Id1/2/3* expression in MS-1 cells (Figures 5A–C). This observation is in striking contrast with the limited increase of *Id2* expression in MS-1 cells exposed for 3 h to TGF-β2, and with the unaffected *Id1* and *Id2* gene expression levels in these cells even after 6 and 24 h treatments with TGF-β2 (Figures 5A–C). In contrast to its marginal effect on *Id1* and *Id2* expression, albeit to low levels, TGF-β2 did augment *Id3* expression in MS-1 cells after 3, 6, and 24 h incubations. These findings on the differential effects of TGF-β2 and BMP9 on the expression of ID proteins in MS-1 cells were generically observed in 2H11 cells as well (Figures 5D–F). The upregulation of *Id1/2/3* by BMP9, and not by TGF-β2, led us to investigate the role of Id proteins as possible negative regulators of the EndMT process.

**FIGURE 6:**
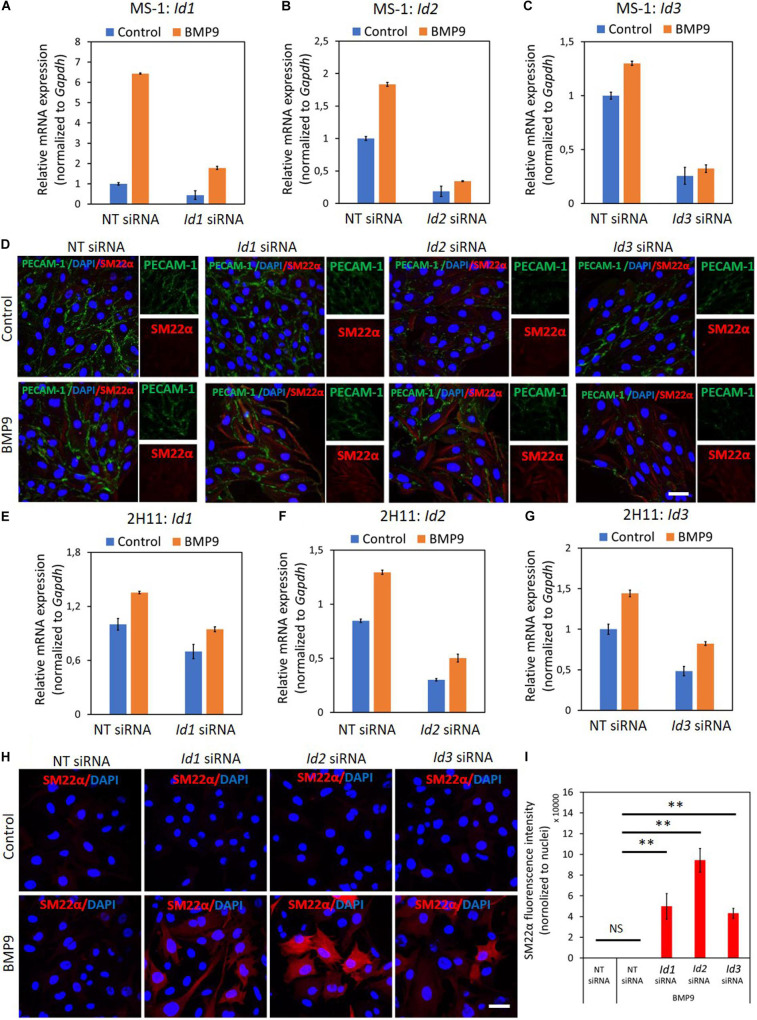
ID proteins are critical in the BMP9-dependent maintenance of endothelial cell phenotypes. **(A–C)** RT-qPCR analysis of the siRNA-mediated silencing of endogenous and BMP9-induced *Id1* **(A)**, *Id2* **(B)** and *Id3* **(C)** mRNA expression in MS-1 cells. Expression levels were normalized to those of the housekeeping gene *Gapdh*. Results are expressed as mean ± SD. Representative results from three independent experiments are shown. **(D)** Fluorescence microscopy analysis of endothelial and mesenchymal markers in MS-1 cells. Non-targeting knockdown, *Id1* knockdown, *Id2* knockdown and *Id3* knockdown MS-1 cells were incubated in medium containing BMP9 (5 ng ml^–1^) or medium containing ligand buffer (control) for 3 days. The expression of endothelial cell marker PECAM-1 (green) and mesenchymal cell marker SM22α (red) in nuclei (blue) stained MS-1 cells were assessed by using immunofluorescent staining. Scale bar: 50 μm. **(E–G)** RT-qPCR analysis of the siRNA-mediated silencing of endogenous and BMP9-induced *Id1* **(E)**, *Id2* **(F)**, and *Id3* **(G)** mRNA expression in MS-1 cells. The expression levels were normalized to those of the housekeeping gene *Gapdh*. Results are expressed as mean ± SD. Representative results from three independent experiments are shown. **(H)** Fluorescence microscopy analysis of mesenchymal marker in 2H11 cells. Non-targeting knockdown, *Id1* knockdown, *Id2* knockdown and *Id3* knockdown 2H11 cells were incubated in medium containing BMP9 (5 ng ml^–1^) or medium containing ligand buffer (control) for 3 days. The expression of mesenchymal cell marker SM22α (red) in nuclei (blue) stained 2H11 cells was assessed by using immunofluorescent staining. Scale bar: 50 μm. **(I)** Quantified mean fluorescence intensity of SM22α. At least six representative images from three repeated experiments were quantified. Results are expressed as mean ± SD. NS, not significant; **p < 0.005.

To this end, we transiently knocked-down ID proteins expression by transfecting siRNAs targeting *Id1*, *Id2*, or *Id3*. As shown in Figures 6A–C, both steady-state and BMP9-induced *Id1*, *Id2*, and *Id3* mRNA levels were decreased after separately transfecting siRNA targeting *Id1*, *Id2*, or *Id3* into MS-1 cells. To investigate whether suppressing the expression of a specific *Id* gene influences the expression of the other two *Id* genes, we checked the levels of each of the three Id proteins in MS-1 and 2H11 cells subjected to knockdown of individual *Id* gene transcripts. As shown in Supplementary Figure S16A, *Id2* and *Id3* expression levels were not overtly influenced by knocking down *Id1* expression in MS-1 cells. Likewise, neither *Id1* and *Id3* nor *Id1* and *Id2* expression levels were substantially affected by knocking down *Id2* or *Id3*, respectively, in MS-1 cells (Supplementary Figure S16A). However, in 2H11 cells, inhibiting the expression of one of the *Id* genes slightly downregulate the other two tested *Id* members (Supplementary Figure S16B). We further treated the *Id1*, *Id2*, and *Id3* knocked down MS-1 cells with BMP9 for 3 days and stained these cells for markers associated with EndMT (i.e., PECAM-1 and SM22α). As shown in Figure 6D, whereas BMP9 did not trigger clear marker changes in MS-1 cells transfected with the non-targeting (NT) siRNA control, this ligand did increase the expression of the mesenchymal cell marker SM22α in MS-1 cells knocked down for *Id1*, *Id2*, or *Id3* (Figure 6D). The quantification of the SM22α fluorescence intensity is shown in Supplementary Figure S17A. Significantly, BMP9 had a lesser obvious effect on altering the expression of the endothelial cell marker PECAM-1 in MS-1 cells knocked down for *Id1*, *Id2* or *Id3* when compared to the changes that it triggered in SM22α expression (Figure 6D). The quantification of PECAM-1 fluorescence intensity is shown in Supplementary Figure S17B. Importantly, knocking down of *Id* genes in 2H11 cells (Figures 6E–G) was sufficient for triggering BMP9-induced EndMT, as observed by increased SM22α expression, whereas BMP9 did not upregulate SM22α expression in control cells transfected with NT siRNA (Figures 6H,I). Thus, *Id* depletion facilitates BMP9 in inducing, at least partially, an EndMT response.

To further validate this conclusion, we used the Id chemical inhibitor AGX51, which is known to inhibit the ID1-E47 interaction, leading to ubiquitin-mediated degradation of IDs (Wojnarowicz et al., 2019, 2020). We firstly titrated AGX51 (0–80 μM) in MS-1 and 2H11 cells for 24 h to determine an effective concentration. Significantly, consistent suppression of ID1, ID2, and ID3 levels started to be observed when the cells were treated with ≥ 20 μM of AGX51 (Supplementary Figures S18A,B). However, at these AGX51 concentrations, cells showed obvious signs of toxicity (data not shown). In any case, incubation of MS-1 cells with AGX51 led to a marked decrease in endothelial PECAM-1 marker expression and a concomitant enhancement in mesenchymal SM22α marker expression (Supplementary Figure S18C). Similarly, in the presence of AGX51, 2H11 cells showed a mesenchymal-like phenotype as the amounts of SM22α and F-actin fibers were both strongly increased (Supplementary Figure S18D). These results indicate that inhibition of ID proteins by AGX51 triggers the acquisition of EndMT hallmarks by MS-1 and 2H11 cells.

### ID Proteins Antagonize TGF-β2-Induced EndMT

**FIGURE 7:**
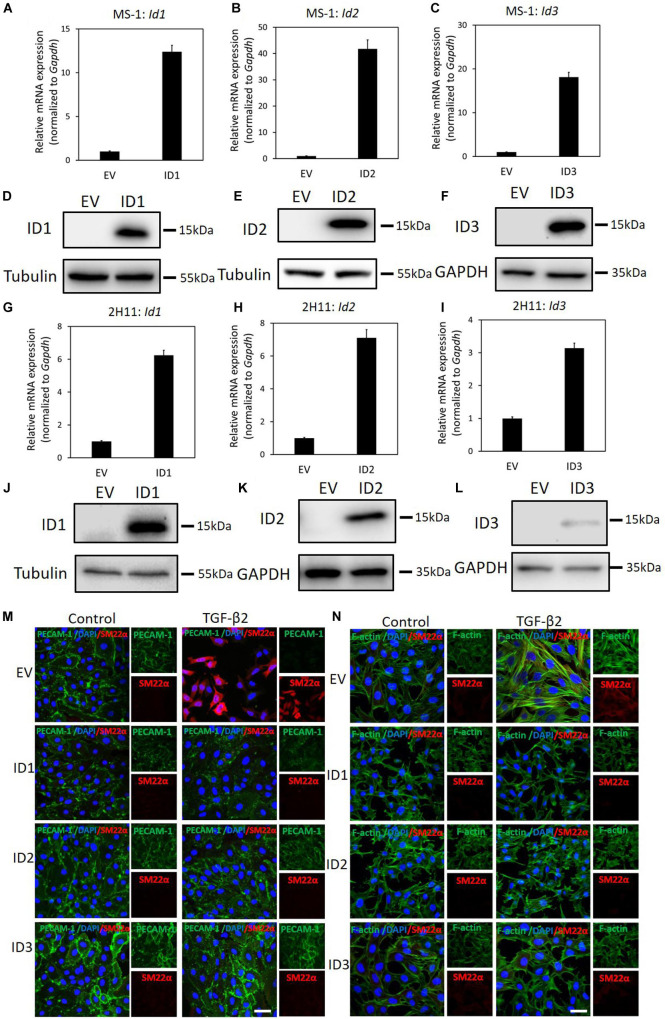
ID proteins antagonize TGF-β2-induced EndMT. **(A–C)** RT-qPCR analysis of lentivirus-mediated ectopic expression of *Id1***(A)**, *Id2* **(B)**, and *Id3* **(C)** mRNA in MS-1 cells. Expression levels were normalized to those of the housekeeping gene *Gapdh*. Results are expressed as mean ± SD. **(D–F)** Western blot analysis of lentivirus-mediated ectopic expression of ID1**(D)**, ID2 **(E)**, and ID3 **(F)** proteins in MS-1 cells. **(G–I)** RT-qPCR analysis of lentivirus-mediated ectopic expression of *Id1***(G)**, *Id2* **(H)**, and *Id3* **(I)** mRNA in 2H11 cells. Expression levels were normalized to those of the housekeeping gene *Gapdh*. Results are expressed as mean ± SD. **(J–L)** Western blot analysis of lentivirus-mediated ectopic expression of ID1**(J)**, ID2 **(K)**, and ID3 **(L)** proteins in 2H11 cells. **(M)** Fluorescence microscopy analysis of endothelial and mesenchymal markers in MS-1 cells. Empty vector (EV) expressing MS-1, ID1-overexpressing, ID2-overexpressing and ID3-overexpressing MS-1 cells were incubated in medium containing TGF-β2 (1 ng ml^–1^) or medium containing ligand buffer (control) for 3 days. The expression of endothelial cell marker PECAM-1 (green) and mesenchymal cell marker SM22α (red) in nuclei (blue) stained MS-1 cells were assessed by using immunofluorescent staining. Scale bar: 50 μm. **(N)** Fluorescence microscopy analysis of mesenchymal markers in 2H11 cells. Empty vector (EV) expressing 2H11, ID1-overexpressing, ID2-overexpressing and ID3-overexpressing 2H11 cells were incubated in medium containing TGF-β2 (1 ng ml^–1^) or medium containing ligand buffer (control) for 3 days. The expression of mesenchymal cell markers F-actin (green) SM22α (red) in nuclei (blue) stained 2H11 cells were assessed by using immunofluorescent staining. Representative results from three independent experiments are shown. Scale bar: 50 μm.

We have demonstrated that genetic and pharmacological inhibition ofID proteins favors BMP9-induced EndMT. Next, we wondered whether upregulation of ID proteins may prevent TGF-β-induced EndMT. To test this, ID1/2/3 were stably expressed in MS-1 and 2H11 cells using lentiviral vectors, as shown by RT-qPCR and western blot analysis (Figures 7A–L). As shown in Figure 7M, upon ectopic expression of ID1, ID2, or ID3, TGF-β2 failed to increase the expression of SM22α, while the expression of PECAM-1 was partially stabilized. The quantification of the fluorescence intensity of SM22α and PECAM-1 is shown in Supplementary Figures S19A,B. This result suggests that constitutive expression of ID1, ID2, or ID3 antagonizes TGF-β2-mediated EndMT in MS-1 cells. Similarly, overexpression of ID1, ID2, or ID3 favored the retention of the endothelial phenotype in 2H11 cells exposed to TGF-β2 (Figure 7N). In Supplementary Figures S19C,D the quantification of the fluorescence intensities corresponding to the F-actin and SM22α markers in 2H11 is shown. Therefore, we conclude that ID proteins predispose endothelial cells to maintain an endothelial phenotype by interfering with EndMT stimuli.

### BMP9 Does Not Antagonize TGF-β2-Induced EndMT

**FIGURE 8:**
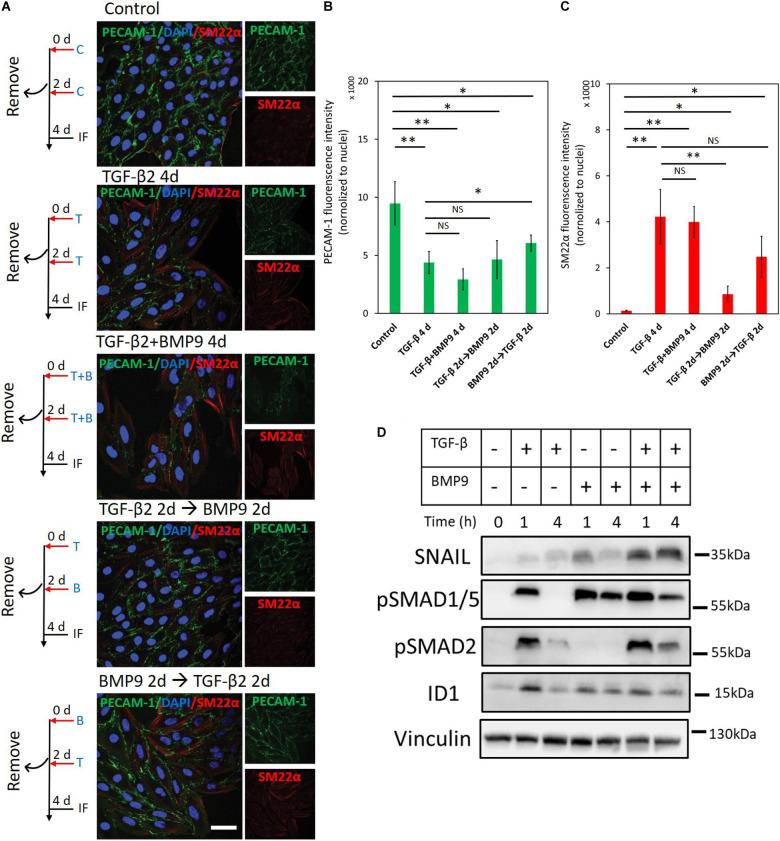
BMP9 does not antagonize TGF-β2-induced EndMT. **(A)** Fluorescence microscopy analysis of endothelial and mesenchymal markers in MS-1 cells. MS-1 cells were incubated in medium containing ligand buffer (control, C) or TGF-β2 (1 ng ml^–1^, T) and BMP9 (5 ng ml^–1^, B) for 4 days, or firstly incubated with TGF-β2 (1 ng ml^–1^) for 2 days and then change to BMP9 (5 ng ml^–1^) for 2 days, or firstly incubated with BMP9 (5 ng ml^–1^) for 2 days and then change to TGF-β2 (1 ng ml^–1^) for 2 days. The expression of the endothelial cell marker PECAM-1 (green) and the mesenchymal cell marker SM22α (red) in nuclei (blue) stained MS-1 cells were assessed by using immunofluorescent staining. Scale bar: 50 μm. **(B,C)** Quantified mean fluorescence intensity of PECAM-1 **(B)** and SM22α **(C)**. At least four representative images from three independent experiments were quantified. Results are expressed as mean ± SD. NS, not significant; *p < 0.05, **p < 0.005. **(D)** Western blot analysis of the effects of TGF-β2 (1 ng ml^–1^) or/and BMP9 (5 ng ml^–1^) on pSmad1/5, pSMAD2, SNAIL, and ID1 proteins expression after 1 and 4 h treatments in MS-1 cells.

Our results indicate that the Ids induced by BMP9 contribute to maintaining an endothelial cell phenotype, while TGF-β2 actives SNAIL to drive endothelial cells toward a fibroblast-like appearance. To investigate the interplay between BMP9 and TGF-β2 in the context of EndMT, we analyzed changes in the expression of endothelial and mesenchymal markers upon concomitant or sequential stimulation of MS-1cells with TGF-β2 and BMP9 (Figures 8A–C). As shown in Figure 8A, after 4 days of TGF-β2 stimulation, the expression of PECAM-1 decreased while SM22α increased, as expected. Interestingly, when the cells were treated with a TGF-β2 and BMP9 combination for 4 days, TGF-β2-induced EndMT was not affected, suggesting that the upregulation of SM22α and the down-regulation of PECAM-1 were not influenced by BMP9 (Figure 8A). Moreover, EndMT still occurred in MS-1 cells upon a treatment of TGF-β2 for 2 days followed by a 2 day BMP9 stimulation or vice versa (Figure 8A). The quantification of fluorescence intensities derived from PECAM-1- and SM22α-directed confocal microscopy analyses of three independent experiments is shown in Figures 8B,C. Thus, 2 days of TGF-β2 treatment is sufficient to induce EndMT with neither the concomitant nor sequential presence of BMP9 affecting this process. To obtain further insights on the non-antagonizing effect of BMP9 on TGF-β2-induced EndMT, we studied SNAIL and ID1 expression changes after exposing MS-1 cells to TGF-β2 and BMP9 either individually or simultaneously (Figure 8D). BMP9 enhanced TGF-β2 activation of the TGF-β signaling pathway, as BMP9 upregulated TGF-β2-induced SMAD2 phosphorylation, when compared to cells exposed exclusively to TGF-β2. Moreover, SNAIL expression was strongly raised in the presence of both BMP9 and TGF-β2 when compared to its expression in cells treated with BMP9 and TGF-β2 individually. Interestingly, the induction of ID1 in combined treatments was similar to that induced by BMP9 only. Quantification of SNAIL, pSMAD2 and ID1 amounts by densitometry analysis of western blots are shown in Supplementary Figures S20A–C. The augmented expression of SNAIL, and retained ID1 expression, might explain why BMP9 fails to inhibit TGF-β2 induced EndMT.

## Discussion

**FIGURE 9:**
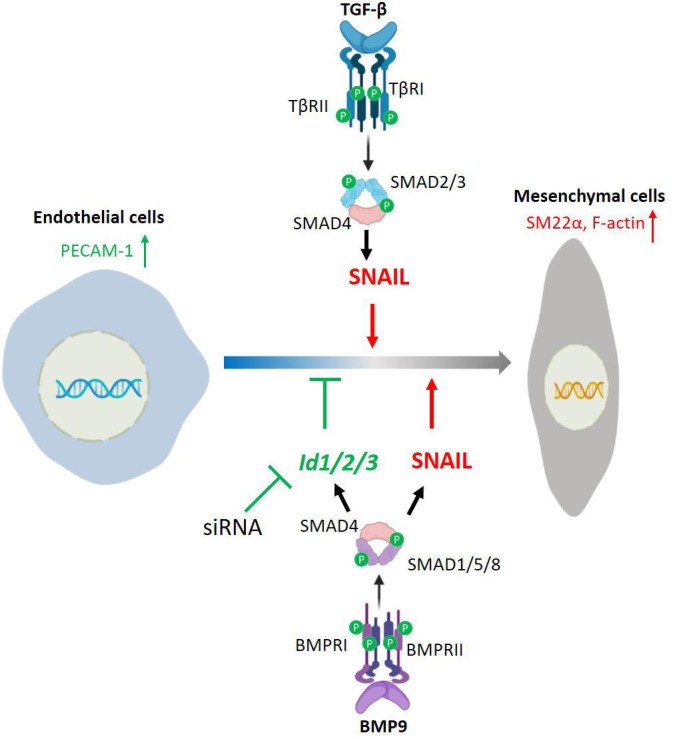
Schematic of a working model by which TGF-β and BMP9 regulate EndMT. TGF-β/SMAD signaling induces EndMT by promoting SNAIL expression. BMP9/SMAD signaling promotes SNAIL and ID1/2/3 expression, and stimulates EndMT only when BMP9-induced expression of *Id1/2/3* is silenced.

Emerging evidence points to a pivotal role of EndMT in both embryonic development and clinical disorders (Ma et al., 2020). Moreover, targeting specific EndMT pathways is also gaining considerable interest for its exploitation in tissue engineering (Hong et al., 2018; Ma et al., 2020). Previous studies have highlighted the role of TGF-β family proteins as the main drivers and regulators of multistep and dynamic EndMT processes. However, in order to target and manipulate EndMT for biomedical applications, a further/deeper understanding of the underlying mechanisms is warranted. In this study, we report that TGF-β2 triggers EndMT in two murine endothelial cell lines, i.e., MS-1 and 2H11. By using CRISPR/Cas9-based gene editing, we generated cell lines knocked-out for either *Snail* or *Slug* that served to demonstrate that SNAIL is required for TGF-β2-induced EndMT. When compared to SNAIL, SLUG had a less effect in the induction of EndMT by TGF-β2 in 2H11 cells. Additionally, we found that while BMP9 strongly induced a burst of SNAIL and SLUG expression, it was nonetheless unable to elicit a substantial EndMT response. Mechanistically, we observed that BMP9-induced ID proteins antagonize EndMT, as inhibition of *Id1*, *Id2*, or *Id3* mRNA expression in ECs enabled BMP9 to trigger EndMT. Moreover, ectopic expression of these ID proteins individually attenuated TGF-β-mediated EndMT. Thus, whereas SNAIL is a key mediator, ID proteins function as gatekeepers of the EndMT process (Figure 9). We further showed that TGF-β2 is a strong inducer of EndMT in MS-1 and 2H11 cells, which is in line with previous reports (Yoshimatsu and Watabe, 2011; Ten Dijke et al., 2012). In contrast to TGF-β2, and similarly to BMP6, BMP9, either failed to induce or prevented EndMT (Figure 1 and Supplementary Figure S2). Related to these findings, it is noteworthy mentioning that Medici and co-workers showed that, like TGF-β2, BMP4 can trigger EndMT in a BMP type I receptor/ALK2-dependent manner in both human umbilical vein endothelial cells (HUVECs) and human cutaneous microvascular endothelial cells (HCMECs) (Medici et al., 2010). BMP7 has been reported to act as an EndMT suppressor and being able to abrogate TGF-β1-induced EndMT in human coronary endothelial cells and cardiac fibrosis (Zeisberg et al., 2007). BMP9 and BMP10 have been shown to mediate the closure of the ductus arteriosus at birth via inducing EndMT (Levet et al., 2015). Since endothelial cells exhibit broad degrees of specialization in different organs (Dejana et al., 2017; Pinto et al., 2018), our results suggest that BMPs regulate EndMT in a cell- and/or tissue-specific manner.

The SNAIL family of transcription factors members have been considered to be key modulators of EndMT processes (Kokudo et al., 2008; Medici et al., 2011; Ma et al., 2020). We found that SNAIL expression was strongly stimulated by TGF-β2 (Figure 2). The genetic depletion of *Snail* in 2H11 and MS-1 cells inhibited EndMT, indicating that SNAIL is essential for TGF-β2-induced EndMT in these cells (Figures 3, 4). Unlike our results, Mihira and colleagues reported that *Snail* inhibition failed to abrogate TGF-β2-induced α-SMA expression and EndMT in MS-1 cells (Mihira et al., 2012). In contrast to the transient siRNA-induced knockdown of *Snail* expression performed by Mihira et al., we permanently ablated *Snail* by CRISPR/Cas9-induced knockout. Together, these data suggest that low levels of SNAIL may be sufficient to mediate EndMT in MS-1 cells. Moreover, we demonstrated that the CRISPR/Cas9-induced depletion of *Slug* could partially inhibit TGF-β2-induced EndMT in 2H11 cells (Figure 4). Our laboratory has previously shown the importance of SLUG in regulating the expression of the endothelial cell marker PECAM-1 and in calcific differentiation of ECs (Sánchez-Duffhues et al., 2015). In the case of the transcription factors *Twist* and *Zeb1*, that are regulated by TGF-β2 and/or BMP9, we were unable to obtain consistent results in MS-1 and 2H11 cells, which might indicate that the function of these two transcription factors in EndMT is not as key as that of SNAIL in most ECs.

When investigating the effect of SNAIL and SLUG through cell proliferation and migration assays, we found that the depletion of SNAIL inhibited the proliferation of MS-1 cells and promoted the proliferation of 2H11 cells (Figures 3B, 4D). This is in line with previous publications in which opposite effects were also reported regarding the role of SNAIL in regulating cell numbers (Yang et al., 2017; Liu et al., 2019). In cell migration assays, we found that SNAIL promoted and failed to promote significant cell motility in MS-1 and 2H11 cells, respectively (Figures 3C, 4E). A number of earlier reports demonstrated that SNAIL enhances the migration of MDA-MB-231 cells (Wu et al., 2009; Ito et al., 2016). Hence, the differential effects of SNAIL and SLUG on cell proliferation and migration might result from different cell origins or expression patterns of other proteins that are alternatively controlled by SNAIL or SLUG deficiency. Further research is warranted to dissect these possibilities.

In contrast to the SNAIL family of transcription factors, ID proteins were identified as EndMT suppressors. Our data demonstrates that the depletion of either *Id1*, *Id2*, or *Id3* was sufficient for letting BMP9 to induce the expression of the mesenchymal cell marker SM22α in both endothelial cell lines (i.e., MS-1 and 2H11) (Figure 6). As corollary, this data indicates that BMP9 promotes the acquisition of EndMT features by MS-1 and 2H11 cells when the expression of ID proteins is dampened. Furthermore, ectopic expression of ID1, ID2 or ID3 prevented the build-up of mesenchymal cell markers in ECs upon TGF-β2 treatment (Figure 7), indicating a critical negative regulatory role of IDs on initiating and/or driving the EndMT process. These results may explain the fact that BMP9 strongly increased SNAIL (and even SLUG) expression yet, it was unable to induce EndMT. Indeed, BMP9 induced robust activation of *Id* gene expression, whose products likely went on to oppose SNAIL- and SLUG-mediated EndMT. Interestingly, in cells incubated with TGF-β2 and BMP9, the latter ligand was unable to inhibit TGF-β2-induced EndMT (Figure 8). This observation may be caused by the augmented TGF-β2-dependent induction of SNAIL expression despite a retained induction of ID expression in response to BMP9.

To the best of our knowledge, there are no previous studies on the effect of IDs on the EndMT process, whilst the role of IDs in EMT remains controversial (Kondo et al., 2004; Hu et al., 2013; Stankic et al., 2013; Castañón et al., 2017). For instance, after dimerizing with E2A, IDs act as negative regulators of EMT by preventing E2A-mediated suppression of epithelial-specific protein expression (Kondo et al., 2004; Stankic et al., 2013). Other studies, however, demonstrated that ID members, in particular ID1, favored the EMT process in tumor cells (Hu et al., 2013; Castañón et al., 2017).

In summary, our work provides new insights into the role of SNAIL and SLUG in EndMT pathways controlled by TGF-β family members. Furthermore, we identified ID proteins ID1, ID2, and ID3 as critical EndMT suppressors. These findings may be further explored to, by taking into account the balance between SNAIL and ID family members, pharmacologically modulate EndMT for scientific or therapeutic purposes. Regarding the latter aspect, our results may contribute to develop novel approaches permitting a precise control over EndMT for the treatment of fibrotic diseases or for devising tissue engineering applications.

## Data Availability Statement

The raw data supporting the conclusions of this article will be made available by the authors, without undue reservation, to any qualified researcher.

## Author Contributions

JM, GZ, and MT performed the experiments. MG designed the gRNA acceptor construct AA19_pLKO.1-puro.U6.sgRNA.*Bve*I-Dys and the gRNA oligos for CRISPR/Cas9-mediated gene knockouts. JM and PD analyzed the data. JM wrote the manuscript. GS-D, MG, and PD edited and critically revised the manuscript. PD conceived and supervised the project. All authors revised the content and approved the final manuscript.

## Conflict of Interest

The authors declare that the research was conducted in the absence of any commercial or financial relationships that could be construed as a potential conflict of interest.

## Supplementary Material

Supplementary Table 1 Primers used for qRT-PCR.

Supplementary Table 2 Gene targets and gRNA oligonucleotides for CRISPR/Cas9-mediated knockout studies.
