# Supplementary material for: TGF-β-Induced Endothelial to Mesenchymal Transition Is Determined by a Balance Between SNAIL and ID Factors
Source: Front Cell Dev Biol. 2021 Feb 12;9:616610. doi: 10.3389/fcell.2021.616610 (PMC7907445; doi:10.3389/fcell.2021.616610)
Supplement: Supplementary Table 1 — Primers used for qRT-PCR. [file Data_Sheet_1.docx]

**SUPPLEMENTARY INFORMATION**

**TGF-β-induced endothelial to mesenchymal transition is determined by a balance between SNAIL and ID factors**

**Jin Ma^1,2^, Gerard van der Zon^1,2^, Manuel A. F. V. Gonçalves^1^, Maarten van Dinther^1,2^, Midory Thorikay^1,2^, Gonzalo Sanchez-Duffhues^1^ and Peter ten Dijke^1,2,*^**

^1^Dept. Cell Chemical Biology, Leiden University Medical Center, 2300 RC Leiden, The Netherlands

^2^Oncode Institute, Leiden University Medical Center, 2300 RC Leiden, The Netherlands

**^*^Correspondence:** P.ten_Dijke@lumc.nl; Tel.: +31-71-526-9271; Fax: +31-71-526-8270


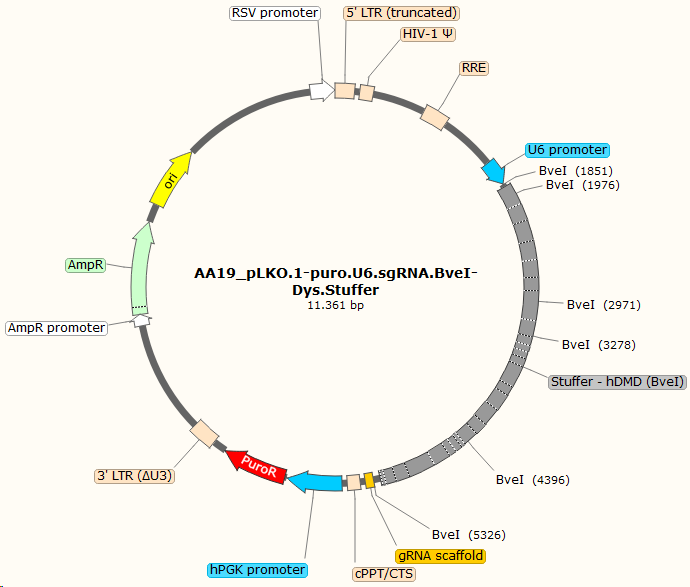


> AA19_pLKO.1-puro.U6.sgRNA.BveI-Dys.Stuffer (11,361 bp)

GGGTCTCTCTGGTTAGACCAGATCTGAGCCTGGGAGCTCTCTGGCTAACTAGGGAACCCACTGCTTAAGCCTCAATAAAG

CTTGCCTTGAGTGCTTCAAGTAGTGTGTGCCCGTCTGTTGTGTGACTCTGGTAACTAGAGATCCCTCAGACCCTTTTAGT

CAGTGTGGAAAATCTCTAGCAGTGGCGCCCGAACAGGGACTTGAAAGCGAAAGGGAAACCAGAGGAGCTCTCTCGACGCA

GGACTCGGCTTGCTGAAGCGCGCACGGCAAGAGGCGAGGGGCGGCGACTGGTGAGTACGCCAAAAATTTTGACTAGCGGA

GGCTAGAAGGAGAGAGATGGGTGCGAGAGCGTCAGTATTAAGCGGGGGAGAATTAGATCGCGATGGGAAAAAATTCGGTT

AAGGCCAGGGGGAAAGAAAAAATATAAATTAAAACATATAGTATGGGCAAGCAGGGAGCTAGAACGATTCGCAGTTAATC

CTGGCCTGTTAGAAACATCAGAAGGCTGTAGACAAATACTGGGACAGCTACAACCATCCCTTCAGACAGGATCAGAAGAA

CTTAGATCATTATATAATACAGTAGCAACCCTCTATTGTGTGCATCAAAGGATAGAGATAAAAGACACCAAGGAAGCTTT

AGACAAGATAGAGGAAGAGCAAAACAAAAGTAAGACCACCGCACAGCAAGCGGCCGCTGATCTTCAGACCTGGAGGAGGA

GATATGAGGGACAATTGGAGAAGTGAATTATATAAATATAAAGTAGTAAAAATTGAACCATTAGGAGTAGCACCCACCAA

GGCAAAGAGAAGAGTGGTGCAGAGAGAAAAAAGAGCAGTGGGAATAGGAGCTTTGTTCCTTGGGTTCTTGGGAGCAGCAG

GAAGCACTATGGGCGCAGCGTCAATGACGCTGACGGTACAGGCCAGACAATTATTGTCTGGTATAGTGCAGCAGCAGAAC

AATTTGCTGAGGGCTATTGAGGCGCAACAGCATCTGTTGCAACTCACAGTCTGGGGCATCAAGCAGCTCCAGGCAAGAAT

CCTGGCTGTGGAAAGATACCTAAAGGATCAACAGCTCCTGGGGATTTGGGGTTGCTCTGGAAAACTCATTTGCACCACTG

CTGTGCCTTGGAATGCTAGTTGGAGTAATAAATCTCTGGAACAGATTTGGAATCACACGACCTGGATGGAGTGGGACAGA

GAAATTAACAATTACACAAGCTTAATACACTCCTTAATTGAAGAATCGCAAAACCAGCAAGAAAAGAATGAACAAGAATT

ATTGGAATTAGATAAATGGGCAAGTTTGTGGAATTGGTTTAACATAACAAATTGGCTGTGGTATATAAAATTATTCATAA

TGATAGTAGGAGGCTTGGTAGGTTTAAGAATAGTTTTTGCTGTACTTTCTATAGTGAATAGAGTTAGGCAGGGATATTCA

CCATTATCGTTTCAGACCCACCTCCCAACCCCGAGGGGACCCGACAGGCCCGAAGGAATAGAAGAAGAAGGTGGAGAGAG

AGACAGAGACAGATCCATTCGATTAGTGAACGGATCTCGACGGTATCGATCACGAGACTAGCCTCGAGCGGCCGCCCCCT

TCACCGAGGGCCTATTTCCCATGATTCCTTCATATTTGCATATACGATACAAGGCTGTTAGAGAGATAATTGGAATTAAT

TTGACTGTAAACACAAAGATATTAGTACAAAATACGTGACGTAGAAAGTAATAATTTCTTGGGTAGTTTGCAGTTTTAAA

ATTATGTTTTAAAATGGACTATCATATGCTTACCGTAACTTGAAAGTATTTCGATTTCTTGGCTTTATATATCTTGTGGA

AAGGACGAAACACCGGTGTGCAGGTGAGTGATCAATTCAGAATCAGTGGGATGAAGTACAAGAACACCTTCAGAACCGGA

GGCAACAGTTGAATGAAATGTTAAAGGATTCAACACAATGGCTGGAAGCTAAGGAAGAAGCTGAGCAGGTCTTAGGACAG

GCCAGAGCCAAGCTTGAGTCATGGAAGGAGGGTCCCTATACAGTAGATGCAATCCAAAAGAAAATCACAGAAACCAAGCA

GTTGGCCAAAGACCTCCGCCAGTGGCAGACAAATGTAGATGTGGCAAATGACTTGGCCCTGAAACTTCTCCGGGATTATT

CTGCAGATGATACCAGAAAAGTCCACATGATAACAGAGAATATCAATGCCTCTTGGAGAAGCATTCATAAAAGGGTGAGT

GAGCGAGAGGCTGCTTTGGAAGAAACTCATAGATTACTGCAACAGTTCCCCCTGGACCTGGAAAAGTTTCTTGCCTGGCT

TACAGAAGCTGAAACAACTGCCAATGTCCTACAGGATGCTACCCGTAAGGAAAGGCTCCTAGAAGACTCCAAGGGAGTAA

AAGAGCTGATGAAACAATGGCAAGACCTCCAAGGTGAAATTGAAGCTCACACAGATGTTTATCACAACCTGGATGAAAAC

AGCCAAAAAATCCTGAGATCCCTGGAAGGTTCCGATGATGCAGTCCTGTTACAAAGACGTTTGGATAACATGAACTTCAA

GTGGAGTGAACTTCGGAAAAAGTCTCTCAACATTAGGTCCCATTTGGAAGCCAGTTCTGACCAGTGGAAGCGTCTGCACC

TTTCTCTGCAGGAACTTCTGGTGTGGCTACAGCTGAAAGATGATGAATTAAGCCGGCAGGCACCTATTGGAGGCGACTTT

CCAGCAGTTCAGAAGCAGAACGATGTACATAGGGCCTTCAAGAGGGAATTGAAAACTAAAGAACCTGTAATCATGAGTAC

TCTTGAGACTGTACGAATATTTCTGACAGAGCAGCCTTTGGAAGGACTAGAGAAACTCTACCAGGAGCCCAGAGAGCTGC

CTCCTGAGGAGAGAGCCCAGAATGTCACTCGGCTTCTACGAAAGCAGGCTGAGGAGGTCAATACTGAGTGGGAAAAATTG

AACCTGCACTCCGCTGACTGGCAGAGAAAAATAGATGAGACCCTTGAAAGACTCCAGGAACTTCAAGAGGCCACGGATGA

GCTGGACCTCAAGCTGCGCCAAGCTGAGGTGATCAAGGGATCCTGGCAGCCCGTGGGCGATCTCCTCATTGACTCTCTCC

AAGATCACCTCGAGAAAGTCAAGGCACTTCGAGGAGAAATTGCGCCTCTGAAAGAGAACGTGAGCCACGTCAATGACCTT

GCTCGCCAGCTTACCACTTTGGGCATTCAGCTCTCACCGTATAACCTCAGCACTCTGGAAGACCTGAACACCAGATGGAA

GCTTCTGCAGGTGGCCGTCGAGGACCGAGTCAGGCAGCTGCATGAAGCCCACAGGGACTTTGGTCCAGCATCTCAGCACT

TTCTTTCCACGTCTGTCCAGGGTCCCTGGGAGAGAGCCATCTCGCCAAACAAAGTGCCCTACTATATCAACCACGAGACT

CAAACAACTTGCTGGGACCATCCCAAAATGACAGAGCTCTACCAGTCTTTAGCTGACCTGAATAATGTCAGATTCTCAGC

TTATAGGACTGCCATGAAACTCCGAAGACTGCAGAAGGCCCTTTGCTTGGATCTCTTGAGCCTGTCAGCTGCATGTGATG

CCTTGGACCAGCACAACCTCAAGCAAAATGACCAGCCCATGGATATCCTGCAGATTATTAATTGTTTGACCACTATTTAT

GACCGCCTGGAGCAAGAGCACAACAATTTGGTCAACGTCCCTCTCTGCGTGGATATGTGTCTGAACTGGCTGCTGAATGT

TTATGATACGGGACGAACAGGGAGGATCCGTGTCCTGTCTTTTAAAACTGGCATCATTTCCCTGTGTAAAGCACATTTGG

AAGACAAGTACAGATACCTTTTCAAGCAAGTGGCAAGTTCAACAGGATTTTGTGACCAGCGCAGGCTGGGCCTCCTTCTG

CATGATTCTATCCAAATTCCAAGACAGTTGGGTGAAGTTGCATCCTTTGGGGGCAGTAACATTGAGCCAAGTGTCCGGAG

CTGCTTCCAATTTGCTAATAATAAGCCAGAGATCGAAGCGGCCCTCTTCCTAGACTGGATGAGACTGGAACCCCAGTCCA

TGGTGTGGCTGCCCGTCCTGCACAGAGTGGCTGCTGCAGAAACTGCCAAGCATCAGGCCAAATGTAACATCTGCAAAGAG

TGTCCAATCATTGGATTCAGGTACAGGAGTCTAAAGCACTTTAATTATGACATCTGCCAAAGCTGCTTTTTTTCTGGTCG

AGTTGCAAAAGGCCATAAAATGCACTATCCCATGGTGGAATATTGCACTCCGACTACATCAGGAGAAGATGTTCGAGACT

TTGCCAAGGTACTAAAAAACAAATTTCGAACCAAAAGGTATTTTGCGAAGCATCCCCGAATGGGCTACCTGCCAGTGCAG

ACTGTCTTAGAGGGGGACAACATGGAAACTCCCGTTACTCTGATCAACTTCTGGCCAGTAGATTCTGCGCCTGCCTCGTC

CCCTCAGCTTTCACACGATGATACTCATTCACGCATTGAACATTATGCTAGCAGGCTAGCAGAAATGGAAAACAGCAATG

GATCTTATCTAAATGATAGCATCTCTCCTAATGAGAGCATAGATGATGAACATTTGTTAATCCAGCATTACTGCCAAAGT

TTGAACCAGGACTCCCCCCTGAGCCAGCCTCGTAGTCCTGCCCAGATCTTGATTTCCTTAGAGAGTGAGGAAAGAGGGGA

GCTAGAGAGAATCCTAGCAGATCTTGAGGAAGAAAACAGGAATCTGCAAGCAGAATATGACCGTCTAAAGCAGCAGCACG

AACATAAAGGCCTGTCCCCACTGCCGTCCCCTCCTGAAATGATGCCCACCTCTCCCCAGAGTCCCCGGGATGCTGAGCTC

ATTGCTGAGGCCAAGCTACTGCGTCAACACAAAGGCCGCCTGGAAGCCAGGATGCAAATCCTGGAAGACCACAATAAACA

GCTGGAGTCACAGTTACACAGGCTAAGGCAGCTGCTGGAGCAACCCCAGGCAGAGGCCAAAGTGAATGGCACAACGGTGT

CCTCTCCTTCTACCTCTCTACAGAGGTCCGACAGCAGTCAGCCTATGCTGCTCCGAGTGGTTGGCAGTCAAACTTCGGAC

TCCATGGGTGAGGAAGATCTTCTCAGTCCTCCCCAGGACACAAGCACAGGGTTAGAGGAGGTGATGGAGCAACTCAACAA

CTCCTTCCCTAGTTCAAGAGGAAGAAATACCCCTGGAAAGCCAATGAGAGAGGACACAATGTAGgaagtctttccacatg

gcggccgcttttttccttAAGCCGAATTGATCAAGAACCTGCTGACGTTTTAGAGCTAGAAATAGCAAGTTAAAATAAGG

CTAGTCCGTTATCAACTTGAAAAAGTGGCACCGAGTCGGTGCTTTTTTTGAATTCTCGACCTCGAGACAAATGGCAGTAT

TCATCCACAATTTTAAAAGAAAAGGGGGGATTGGGGGGTACAGTGCAGGGGAAAGAATAGTAGACATAATAGCAACAGAC

ATACAAACTAAAGAATTACAAAAACAAATTACAAAAATTCAAAATTTTCGGGTTTATTACAGGGACAGCAGAGATCCACT

TTGGCCGCGGCTCGAGGGGGTTGGGGTTGCGCCTTTTCCAAGGCAGCCCTGGGTTTGCGCAGGGACGCGGCTGCTCTGGG

CGTGGTTCCGGGAAACGCAGCGGCGCCGACCCTGGGTCTCGCACATTCTTCACGTCCGTTCGCAGCGTCACCCGGATCTT

CGCCGCTACCCTTGTGGGCCCCCCGGCGACGCTTCCTGCTCCGCCCCTAAGTCGGGAAGGTTCCTTGCGGTTCGCGGCGT

GCCGGACGTGACAAACGGAAGCCGCACGTCTCACTAGTACCCTCGCAGACGGACAGCGCCAGGGAGCAATGGCAGCGCGC

CGACCGCGATGGGCTGTGGCCAATAGCGGCTGCTCAGCAGGGCGCGCCGAGAGCAGCGGCCGGGAAGGGGCGGTGCGGGA

GGCGGGGTGTGGGGCGGTAGTGTGGGCCCTGTTCCTGCCCGCGCGGTGTTCCGCATTCTGCAAGCCTCCGGAGCGCACGT

CGGCAGTCGGCTCCCTCGTTGACCGAATCACCGACCTCTCTCCCCAGGGGGATCCACCGGAGCTTACCATGACCGAGTAC

AAGCCCACGGTGCGCCTCGCCACCCGCGACGACGTCCCCAGGGCCGTACGCACCCTCGCCGCCGCGTTCGCCGACTACCC

CGCCACGCGCCACACCGTCGATCCGGACCGCCACATCGAGCGGGTCACCGAGCTGCAAGAACTCTTCCTCACGCGCGTCG

GGCTCGACATCGGCAAGGTGTGGGTCGCGGACGACGGCGCCGCGGTGGCGGTCTGGACCACGCCGGAGAGCGTCGAAGCG

GGGGCGGTGTTCGCCGAGATCGGCCCGCGCATGGCCGAGTTGAGCGGTTCCCGGCTGGCCGCGCAGCAACAGATGGAAGG

CCTCCTGGCGCCGCACCGGCCCAAGGAGCCCGCGTGGTTCCTGGCCACCGTCGGCGTCTCGCCCGACCACCAGGGCAAGG

GTCTGGGCAGCGCCGTCGTGCTCCCCGGAGTGGAGGCGGCCGAGCGCGCCGGGGTGCCCGCCTTCCTGGAGACCTCCGCG

CCCCGCAACCTCCCCTTCTACGAGCGGCTCGGCTTCACCGTCACCGCCGACGTCGAGGTGCCCGAAGGACCGCGCACCTG

GTGCATGACCCGCAAGCCCGGTGCCTGACGCCCGCCCCACGACCCGCAGCGCCCGACCGAAAGGAGCGCACGACCCCATG

CATCGGTACCTTTAAGACCAATGACTTACAAGGCAGCTGTAGATCTTAGCCACTTTTTAAAAGAAAAGGGGGGACTGGAA

GGGCTAATTCACTCCCAACGAAGACAAGATCTGCTTTTTGCTTGTACTGGGTCTCTCTGGTTAGACCAGATCTGAGCCTG

GGAGCTCTCTGGCTAACTAGGGAACCCACTGCTTAAGCCTCAATAAAGCTTGCCTTGAGTGCTTCAAGTAGTGTGTGCCC

GTCTGTTGTGTGACTCTGGTAACTAGAGATCCCTCAGACCCTTTTAGTCAGTGTGGAAAATCTCTAGCAGTAGTAGTTCA

TGTCATCTTATTATTCAGTATTTATAACTTGCAAAGAAATGAATATCAGAGAGTGAGAGGAACTTGTTTATTGCAGCTTA

TAATGGTTACAAATAAAGCAATAGCATCACAAATTTCACAAATAAAGCATTTTTTTCACTGCATTCTAGTTGTGGTTTGT

CCAAACTCATCAATGTATCTTATCATGTCTGGCTCTAGCTATCCCGCCCCTAACTCCGCCCATCCCGCCCCTAACTCCGC

CCAGTTCCGCCCATTCTCCGCCCCATGGCTGACTAATTTTTTTTATTTATGCAGAGGCCGAGGCCGCCTCGGCCTCTGAG

CTATTCCAGAAGTAGTGAGGAGGCTTTTTTGGAGGCCTAGGGACGTACCCAATTCGCCCTATAGTGAGTCGTATTACGCG

CGCTCACTGGCCGTCGTTTTACAACGTCGTGACTGGGAAAACCCTGGCGTTACCCAACTTAATCGCCTTGCAGCACATCC

CCCTTTCGCCAGCTGGCGTAATAGCGAAGAGGCCCGCACCGATCGCCCTTCCCAACAGTTGCGCAGCCTGAATGGCGAAT

GGGACGCGCCCTGTAGCGGCGCATTAAGCGCGGCGGGTGTGGTGGTTACGCGCAGCGTGACCGCTACACTTGCCAGCGCC

CTAGCGCCCGCTCCTTTCGCTTTCTTCCCTTCCTTTCTCGCCACGTTCGCCGGCTTTCCCCGTCAAGCTCTAAATCGGGG

GCTCCCTTTAGGGTTCCGATTTAGTGCTTTACGGCACCTCGACCCCAAAAAACTTGATTAGGGTGATGGTTCACGTAGTG

GGCCATCGCCCTGATAGACGGTTTTTCGCCCTTTGACGTTGGAGTCCACGTTCTTTAATAGTGGACTCTTGTTCCAAACT

GGAACAACACTCAACCCTATCTCGGTCTATTCTTTTGATTTATAAGGGATTTTGCCGATTTCGGCCTATTGGTTAAAAAA

TGAGCTGATTTAACAAAAATTTAACGCGAATTTTAACAAAATATTAACGCTTACAATTTAGGTGGCACTTTTCGGGGAAA

TGTGCGCGGAACCCCTATTTGTTTATTTTTCTAAATACATTCAAATATGTATCCGCTCATGAGACAATAACCCTGATAAA

TGCTTCAATAATATTGAAAAAGGAAGAGTATGAGTATTCAACATTTCCGTGTCGCCCTTATTCCCTTTTTTGCGGCATTT

TGCCTTCCTGTTTTTGCTCACCCAGAAACGCTGGTGAAAGTAAAAGATGCTGAAGATCAGTTGGGTGCACGAGTGGGTTA

CATCGAACTGGATCTCAACAGCGGTAAGATCCTTGAGAGTTTTCGCCCCGAAGAACGTTTTCCAATGATGAGCACTTTTA

AAGTTCTGCTATGTGGCGCGGTATTATCCCGTATTGACGCCGGGCAAGAGCAACTCGGTCGCCGCATACACTATTCTCAG

AATGACTTGGTTGAGTACTCACCAGTCACAGAAAAGCATCTTACGGATGGCATGACAGTAAGAGAATTATGCAGTGCTGC

CATAACCATGAGTGATAACACTGCGGCCAACTTACTTCTGACAACGATCGGAGGACCGAAGGAGCTAACCGCTTTTTTGC

ACAACATGGGGGATCATGTAACTCGCCTTGATCGTTGGGAACCGGAGCTGAATGAAGCCATACCAAACGACGAGCGTGAC

ACCACGATGCCTGTAGCAATGGCAACAACGTTGCGCAAACTATTAACTGGCGAACTACTTACTCTAGCTTCCCGGCAACA

ATTAATAGACTGGATGGAGGCGGATAAAGTTGCAGGACCACTTCTGCGCTCGGCCCTTCCGGCTGGCTGGTTTATTGCTG

ATAAATCTGGAGCCGGTGAGCGTGGGTCTCGCGGTATCATTGCAGCACTGGGGCCAGATGGTAAGCCCTCCCGTATCGTA

GTTATCTACACGACGGGGAGTCAGGCAACTATGGATGAACGAAATAGACAGATCGCTGAGATAGGTGCCTCACTGATTAA

GCATTGGTAACTGTCAGACCAAGTTTACTCATATATACTTTAGATTGATTTAAAACTTCATTTTTAATTTAAAAGGATCT

AGGTGAAGATCCTTTTTGATAATCTCATGACCAAAATCCCTTAACGTGAGTTTTCGTTCCACTGAGCGTCAGACCCCGTA

GAAAAGATCAAAGGATCTTCTTGAGATCCTTTTTTTCTGCGCGTAATCTGCTGCTTGCAAACAAAAAAACCACCGCTACC

AGCGGTGGTTTGTTTGCCGGATCAAGAGCTACCAACTCTTTTTCCGAAGGTAACTGGCTTCAGCAGAGCGCAGATACCAA

ATACTGTTCTTCTAGTGTAGCCGTAGTTAGGCCACCACTTCAAGAACTCTGTAGCACCGCCTACATACCTCGCTCTGCTA

ATCCTGTTACCAGTGGCTGCTGCCAGTGGCGATAAGTCGTGTCTTACCGGGTTGGACTCAAGACGATAGTTACCGGATAA

GGCGCAGCGGTCGGGCTGAACGGGGGGTTCGTGCACACAGCCCAGCTTGGAGCGAACGACCTACACCGAACTGAGATACC

TACAGCGTGAGCTATGAGAAAGCGCCACGCTTCCCGAAGGGAGAAAGGCGGACAGGTATCCGGTAAGCGGCAGGGTCGGA

ACAGGAGAGCGCACGAGGGAGCTTCCAGGGGGAAACGCCTGGTATCTTTATAGTCCTGTCGGGTTTCGCCACCTCTGACT

TGAGCGTCGATTTTTGTGATGCTCGTCAGGGGGGCGGAGCCTATGGAAAAACGCCAGCAACGCGGCCTTTTTACGGTTCC

TGGCCTTTTGCTGGCCTTTTGCTCACATGTTCTTTCCTGCGTTATCCCCTGATTCTGTGGATAACCGTATTACCGCCTTT

GAGTGAGCTGATACCGCTCGCCGCAGCCGAACGACCGAGCGCAGCGAGTCAGTGAGCGAGGAAGCGGAAGAGCGCCCAAT

ACGCAAACCGCCTCTCCCCGCGCGTTGGCCGATTCATTAATGCAGCTGGCACGACAGGTTTCCCGACTGGAAAGCGGGCA

GTGAGCGCAACGCAATTAATGTGAGTTAGCTCACTCATTAGGCACCCCAGGCTTTACACTTTGGTAATGACTCCAACTTA

TTGATAGTGTTTTATGTTCAGATAATGCCCGATGACTTTGTCATGCAGCTCCACCGATTTTGAGAACGACAGCGACTTCC

GTCCCAGCCGTGCCAGGTGCTGCCTCAGATTCAGGTTATGCCGCTCAATTCGCTGCGTATATCGCTTGCTGATTACGTGC

AGCTTTCCCTTCAGGCGGGATTCATACAGCGGCCAGCCATCCGTCATCCATATCACCACGTCAAAGGGTGACAGCAGGCT

CATAAGACGCCCCAGCGTCGCCATAGTGCGTTCACCGAATACGTGCGCAACAACCGTCTTCCGGAGACTGTCATACGCGT

AAAACAGCCAGCGCTGGCGCGATTTAGCCCCGACATAGCCCCACTGTTCGTCCATTTCCGCGCAGACGATGACGTCACTG

CCCGGCTGTATGCGCGAGGTTACCGACTGCGGCCTGAGTTTTTTAAGTGACGTAAAATCGTGTTGAGGCCAACGCCCATA

ATGCGGGCTGTTGCCCGGCATCCAACGCCATTCATGGCCATATCAATGATTTTCTGGTGCGTACCGGGTTGAGAAGCGGT

GTAAGTGAACTGCAGTTGCCATGTTTTACGGCAGTGAGAGCAGAGATAGCGCTGATGTCCGGCGGTGCTTTTGCCGTTAC

GCACCACCCCGTCAGTAGCTGAACAGGAGGGACAGCTGATAGAAACAGAAGCCACTGGAGCACCTCAAAAACACCATCAT

ACACTAAATCAGTAAGTTGGCAGCATCACCTTACACTTTATGCTTCCGGCTCGTATGTTGTGTGGAATTGTGAGCGGATA

ACAATTTCACACAGGAAACAGCTATGACCATGATTACGCCAAGCGCGCAATTAACCCTCACTAAAGGGAACAAAAGCTGG

AGCTGCAAGCTTAATGTAGTCTTATGCAATACTCTTGTAGTCTTGCAACATGGTAACGATGAGTTAGCAACATGCCTTAC

AAGGAGAGAAAAAGCACCGTGCATGCCGATTGGTGGAAGTAAGGTGGTACGATCGTGCCTTATTAGGAAGGCAACAGACG

GGTCTGACATGGATTGGACGAACCACTGAATTGCCGCATTGCAGAGATATTGTATTTAAGTGCCTAGCTCGATACATAAA

C

**Figure S1** Map and sequence of AA19_pLKO.1-puro.U6.sgRNA.*Bve*I-Dys.Stuffer**.** RSV promoter, Rous sarcoma virus promoter; 5’ LTR (truncated), shortened HIV-1 5’ long terminal repeat (LTR); HIV-1 Ψ, HIV-1 packaging signal; RRE, Rev-responsive element; U6, human *U6* promoter; Stuffer-hDMD (*Bve*I), DNA segment from the human dystrophin-coding sequence; gRNA scaffold, DNA portion coding for the invariant gRNA scaffold; cPPT, HIV-1 central polypurine tract; hPGK-1 promoter, human *phosphoglycerate kinase 1* gene regulatory elements; PuroR, *pac* gene from *Streptomyces* conferring resistance to puromycin; 3’ LTR (ΔU3), self-inactivating 3’ HIV-1 LTR; AmpR promoter and AmpR, *β-lactamase* resistance gene promoter and coding sequence, respectively; ori, prokaryotic pBR322 origin of replication.


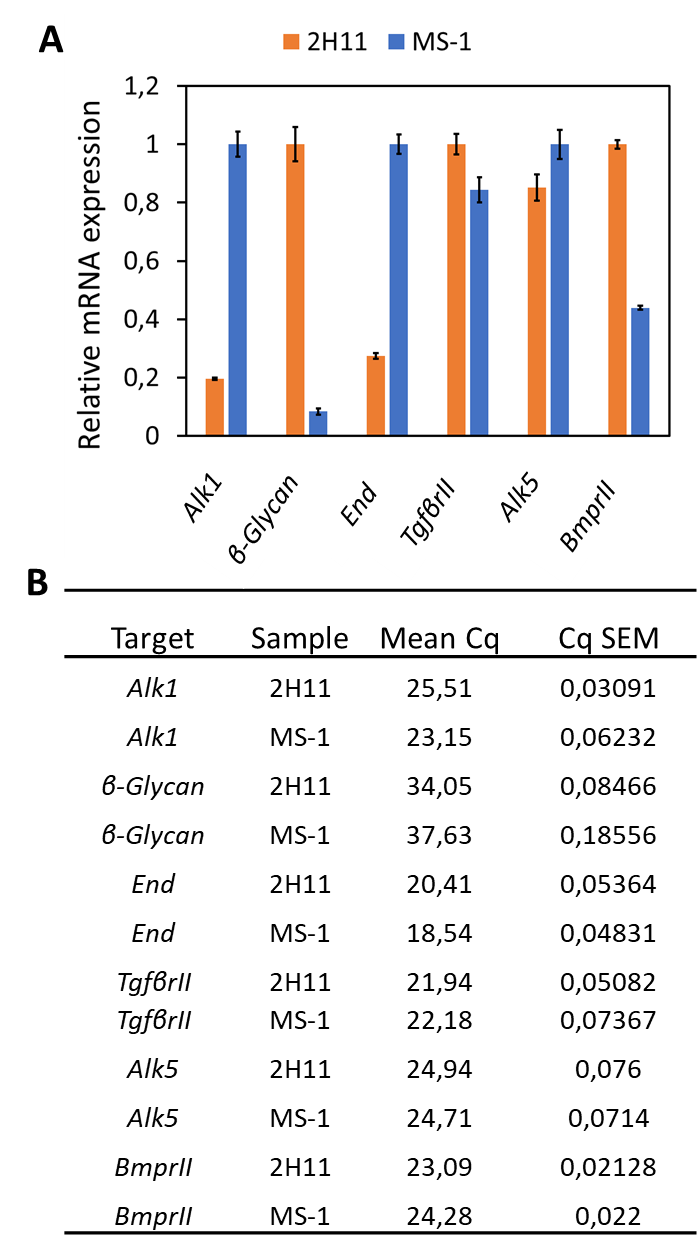


**Figure S2.** **(A)** RT-qPCR analysis of the *Alk1*, *β-Glycan*, *Endoglin*, *TgfβrII*, *Alk5* and *BmprII* expression in MS-1 and 2H11 cells. Expression levels were normalized to that of the housekeeping gene *Gapdh*. (**B**) Cq values of the receptors expression in MS-1 and 2H11 cells as obtained from qRT-PCR analysis. Results from three independent experiments are shown.


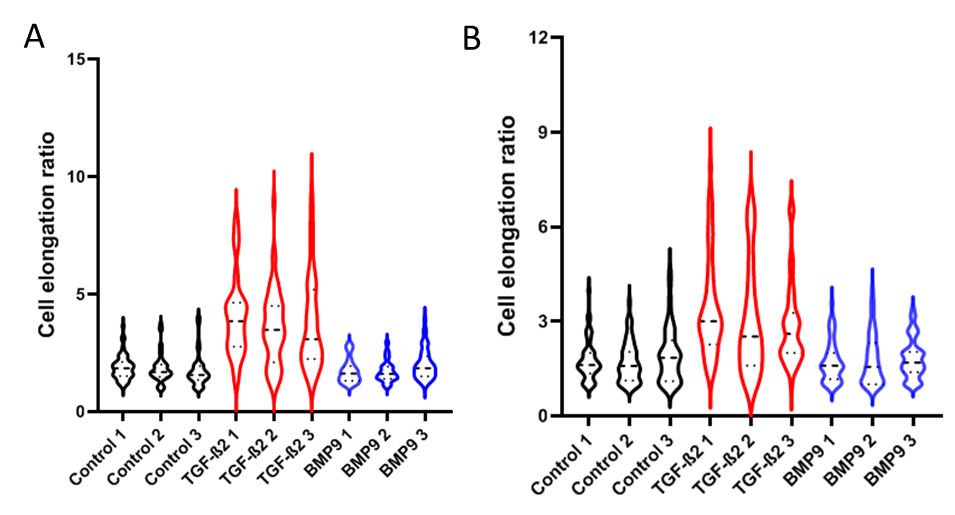


**Figure S3** TGF-β2 induces cell morphology changes compatible with EndMT whilst BMP9 does not. Assessing cell morphological changes induced by TGF-β2 and BMP9 shown in Figure 1A. Quantification of the cell elongation ratio using brightfield microscopy images of MS-1 cells (**A**) or 2H11 (**B**) after treatment with vehicle control, TGF-β2 (1 ng ml^-1^) or BMP9 (5 ng ml^-1^) for 3 days. Results from three independent experiments are shown (labelled with 1, 2 and 3).


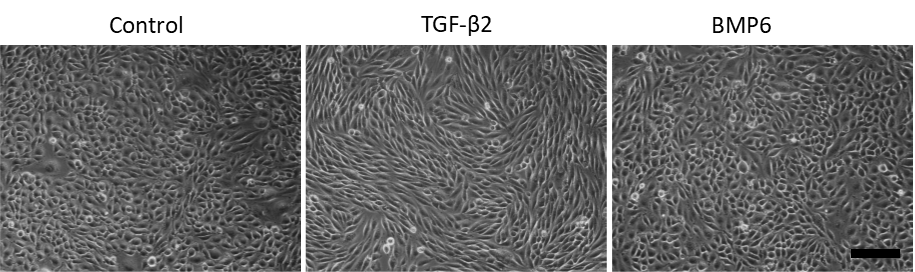


**Figure S4** TGF-β2 induces EndMT whilst BMP6 does not. Assessing cell morphological changes induced by TGF-β2 and BMP6. Brightfield microscopy images of MS-1 cells showing distinct cell morphologies (i.e. cobblestone or fibroblast-like) after treatment with vehicle control, TGF-β2 (1 ng ml^-1^) or BMP6 (50 ng ml^-1^) treatments for 3 days. The experiments were repeated at least three times. Scale bar: 200 μm.


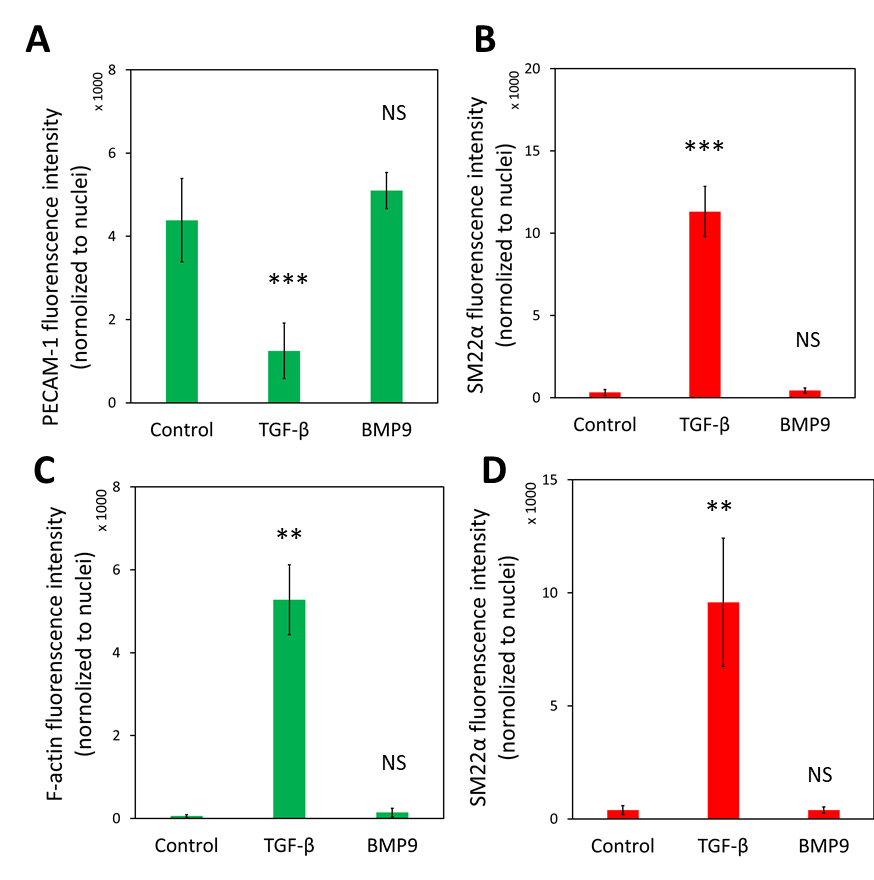


**Figure S5** TGF-β2 induces EndMT whilst BMP9 does not. **(A-B)** Mean fluorescence intensity of PECAM-1 (A) and SM22α (**B**) after treated with vehicle control, TGF-β (1 ng ml^-1^) or BMP9 (5 ng ml^-1^) for 3 days in MS-1 cells. **(C-D)** Mean fluorescence intensity of F-actin (**C**) and SM22α (**D**) after treated with TGF-β (1 ng ml^-1^) or BMP9 (5 ng ml^-1^) for 3 days in 2H11 cells. shown. At least six representative images from three independent experiments were quantified. Results are expressed as mean ± SD. NS, not significant; ***p* < 0.005, ****p* < 0.001.


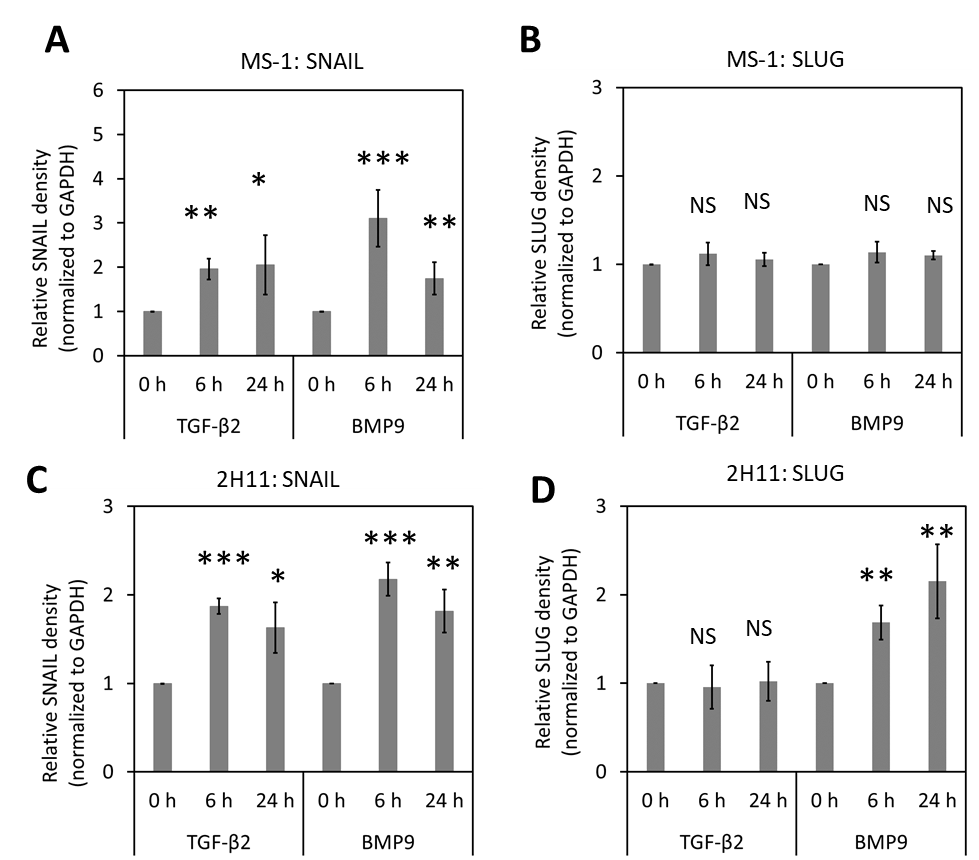


**Figure S6** Effects of TGF-β2 and BMP9 on SNAIL and SLUG protein expression. **(A-B)** Quantified Snail (**A**) and Slug (**B**) expression after TGF-β2 (1 ng ml^-1^) and BMP9 (5 ng ml^-1^) stimulation for 6 h and 24 h in MS-1 cells. GAPDH was used as a loading control. Results from at least three independent experiments are shown. **(C-D)** Quantified Snail (**C**) and Slug (**D**) expression after TGF-β2 (1 ng ml^-1^) and BMP9 (5 ng ml^-1^) stimulation for 6 h and 24 h in 2H11 cells. GAPDH was used as a loading control. Results from at least three independent experiments were quantified. Results are expressed as mean ± SD. NS, not significant; **p* < 0.05, ***p* < 0.005, ****p* < 0.001.


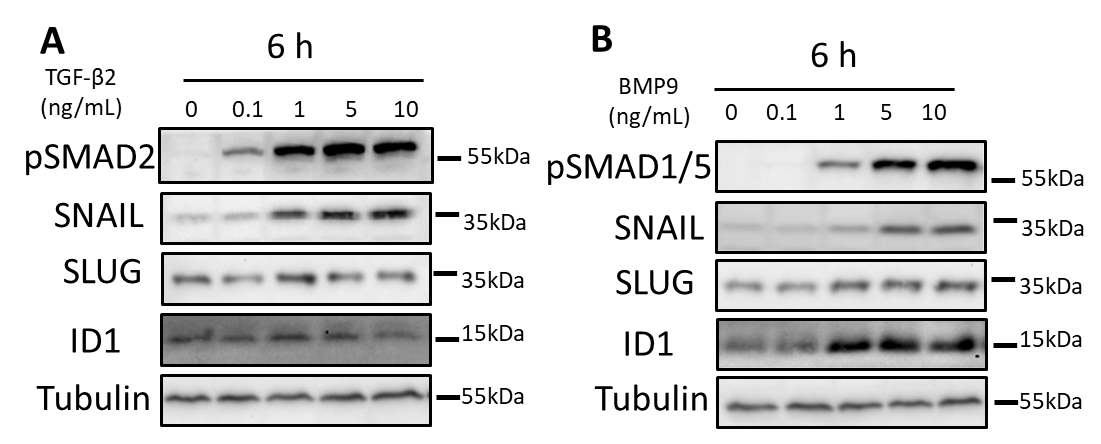


**Figure S7** Effects of different TGF-β2 and BMP9 concentrations on SNAIL and SLUG protein expression. **(A)** pSMAD2, SNAIL, SLUG and ID1 expression after 6 h TGF-β2 (1, 0.1, 1, 5, 10 ng ml^-1^) stimulation in MS-1 cells. Tubulin was used as a loading control. **(B)** pSMAD1, SNAIL, SLUG and ID1 expression after 6 h BMP9 (0, 0.1, 1, 5, 10 ng ml^-1^) stimulation for in MS-1 cells. Tubulin was used as a loading control. All the experiments were repeated three times and representative results are shown.


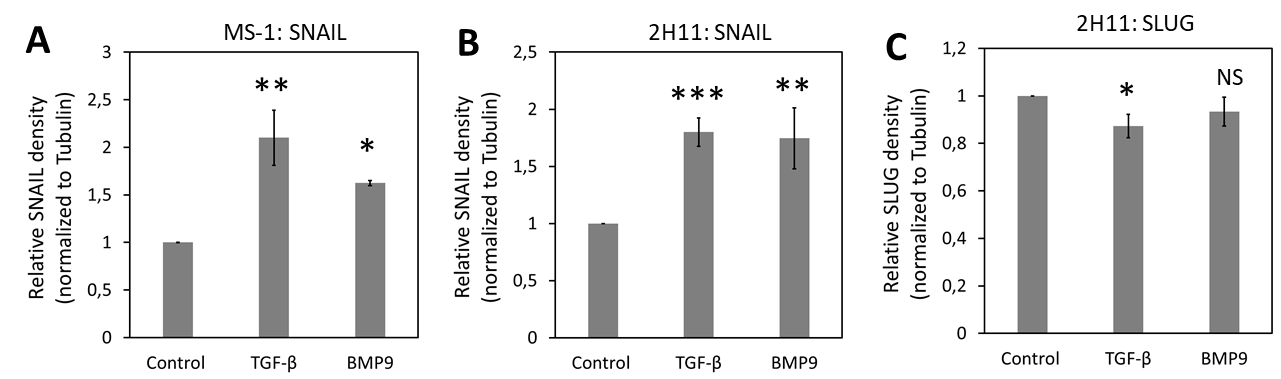


**Figure S8** Effects of TGF-β2 and BMP9 on SNAIL and SLUG protein expression after 3 days. **(A)** Quantified SNAIL expression after TGF-β2 (1 ng ml^-1^) and BMP9 (5 ng ml^-1^) stimulation for 3 days in MS-1 cells. Tubulin was used as a loading control. Results from at least three independent experiments are shown. **(B-C)** Quantified SNAIL (**B**) and SLUG (**C**) expression after TGF-β2 (1 ng ml^-1^) and BMP9 (5 ng ml^-1^) stimulation for 3 days in 2H11 cells. Tubulin was used as a loading control. Results from at least three independent experiments were quantified. Results are expressed as mean ± SD. NS, not significant; **p* < 0.05, ***p* < 0.005, ****p* < 0.001.


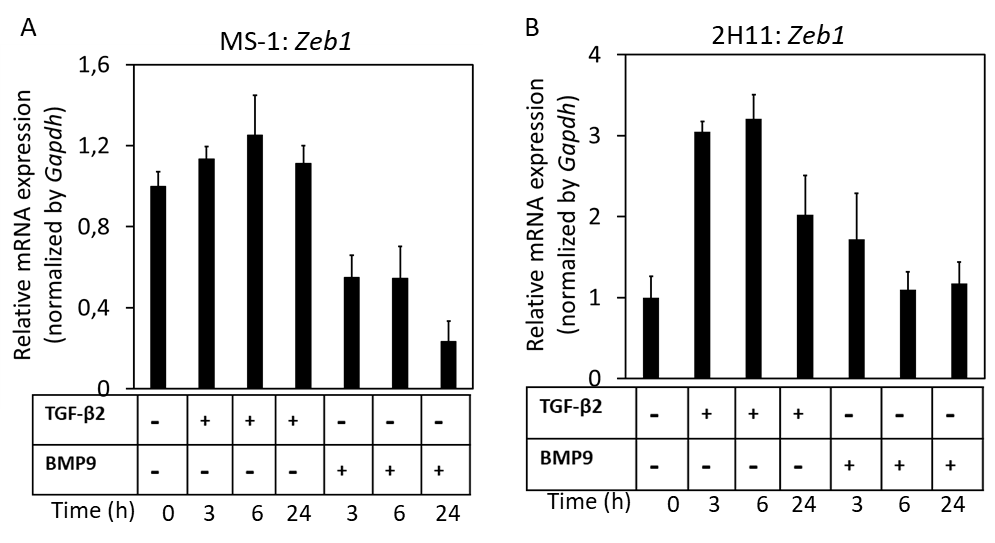


**Figure S9** Effects of TGF-β2 and BMP9 on *Zeb1* expression. RT-qPCR analysis of the effects of TGF-β2 (1 ng ml^-1^), BMP9 (5 ng ml^-1^) or vehicle control on *Zeb1* mRNA expression after 3 h, 6 h and 24 h treatments in MS-1 (**A**) and 2H11 (**B**) cells. All the mRNA expression levels were normalized to the expression of housekeeping gene *Gapdh*. Results are expressed as mean ± SD. Representative results from three independent experiments are shown.


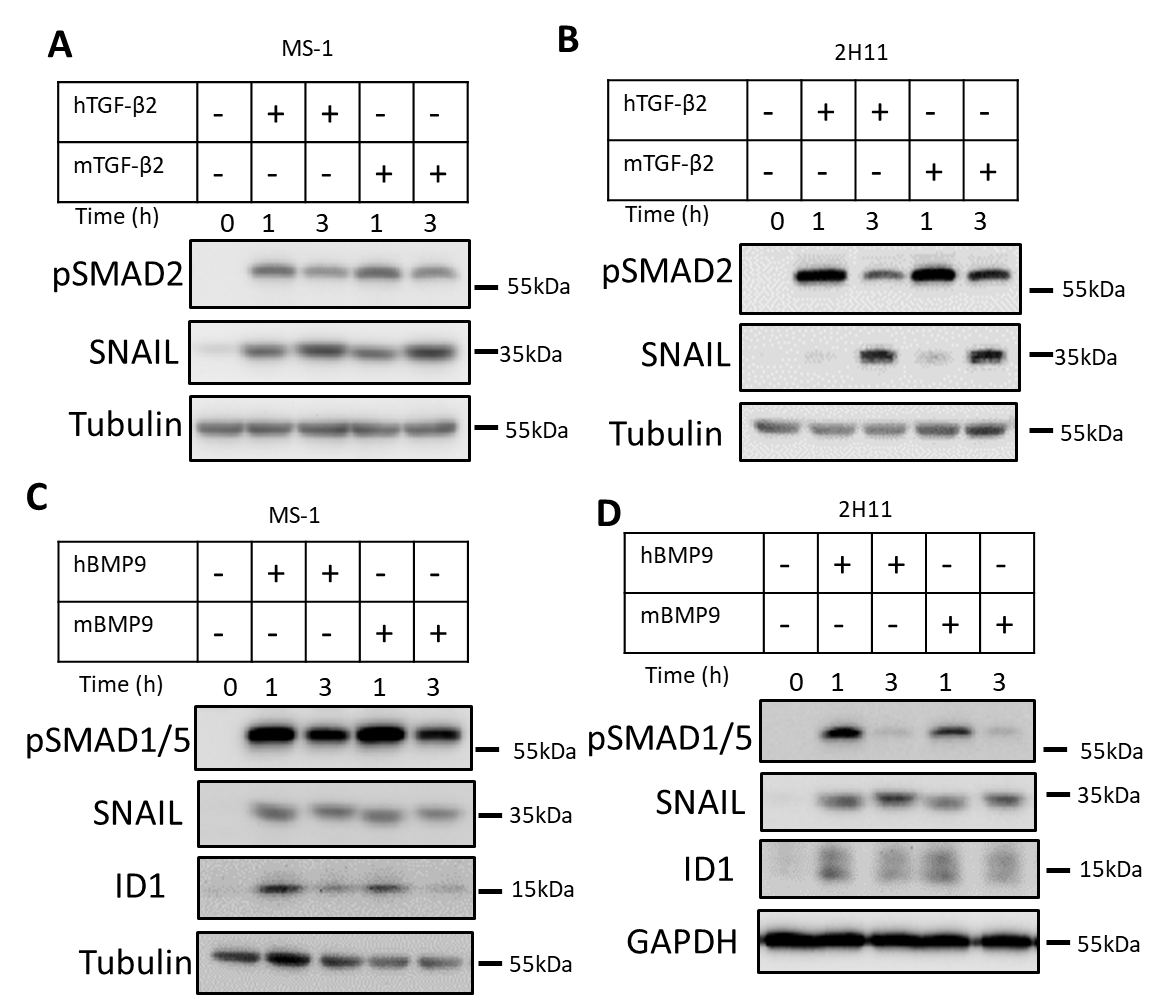


**Figure S10** Comparison of human or mouse TGF-β2 and BMP9 on mouse EC response. **(A-B)** Phosphorylated SMAD2 (pSMAD2) and SNAIL expression after human (h) or mouse (m) TGF-β2 (1 ng ml^-1^) stimulation for 1 and 3 h in MS-1 cells (**A**) and 2H11 cells (**B**). **(C-D)** Phosphorylated SMAD1/5 (P-SMAD1/5), SNAIL and ID1 expression after human or mouse BMP9 (5 ng ml^-1^) stimulation for 1 and 3 h in MS-1 cells (**C**) and 2H11 cells (**D**). Tubulin or GAPDH were used as a loading control. All the experiments were repeated three times and representative experiments are shown.


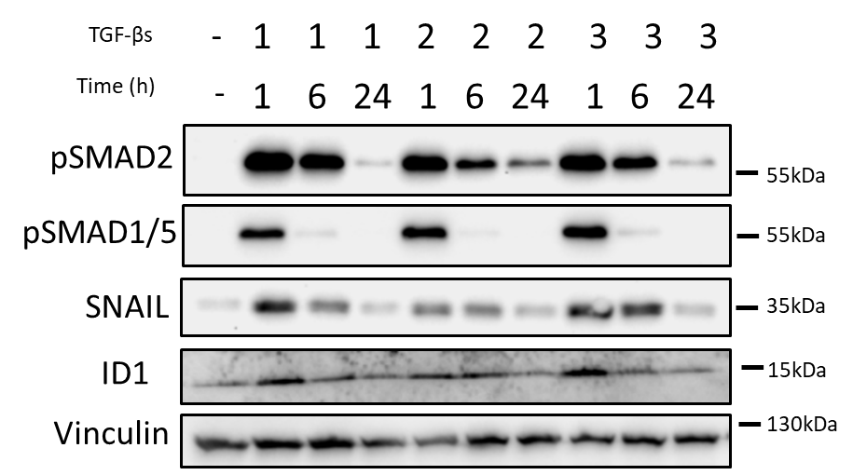


**Figure S11** Effects of three human TGF-β isoforms, i.e. TGF-β1, TGF-β2 and TGF-β3 on MS-1 cells. Phosphorylated SMAD2 (p-SMAD2), Phosphorylated SMAD1/5 (p-SMAD1/5), SNAIL and ID1 expression after TGF-β1, TGF-β2 or TGF-β3 (1 ng ml^-1^) stimulation for 1, 6 and 24 h in MS-1 cells Vinculin was used as a loading control. The experiment was repeated three times and representative results are shown.


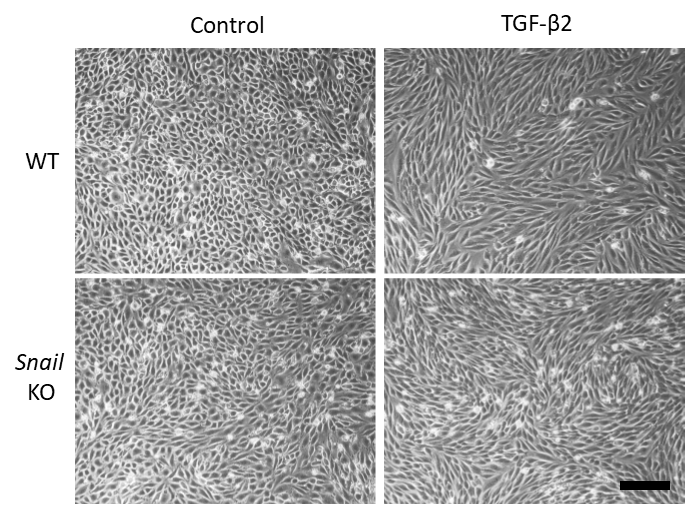


**Figure S12** Depletion of *Snail* weakly attenuated TGF-β2-induced morphology change in MS-1 cells. Brightfield microscopy image analysis of parental MS-1 (upper panel) and *Snail* knockout MS-1 (lower panel) cells in the absence or presence of TGF-β2 (1 ng ml^-1^) for 3 days showed distinct cell morphologies, i.e. cobblestone or fibroblast-like, respectively. The experiments were repeated at least three times. Scale bar: 200 μm.


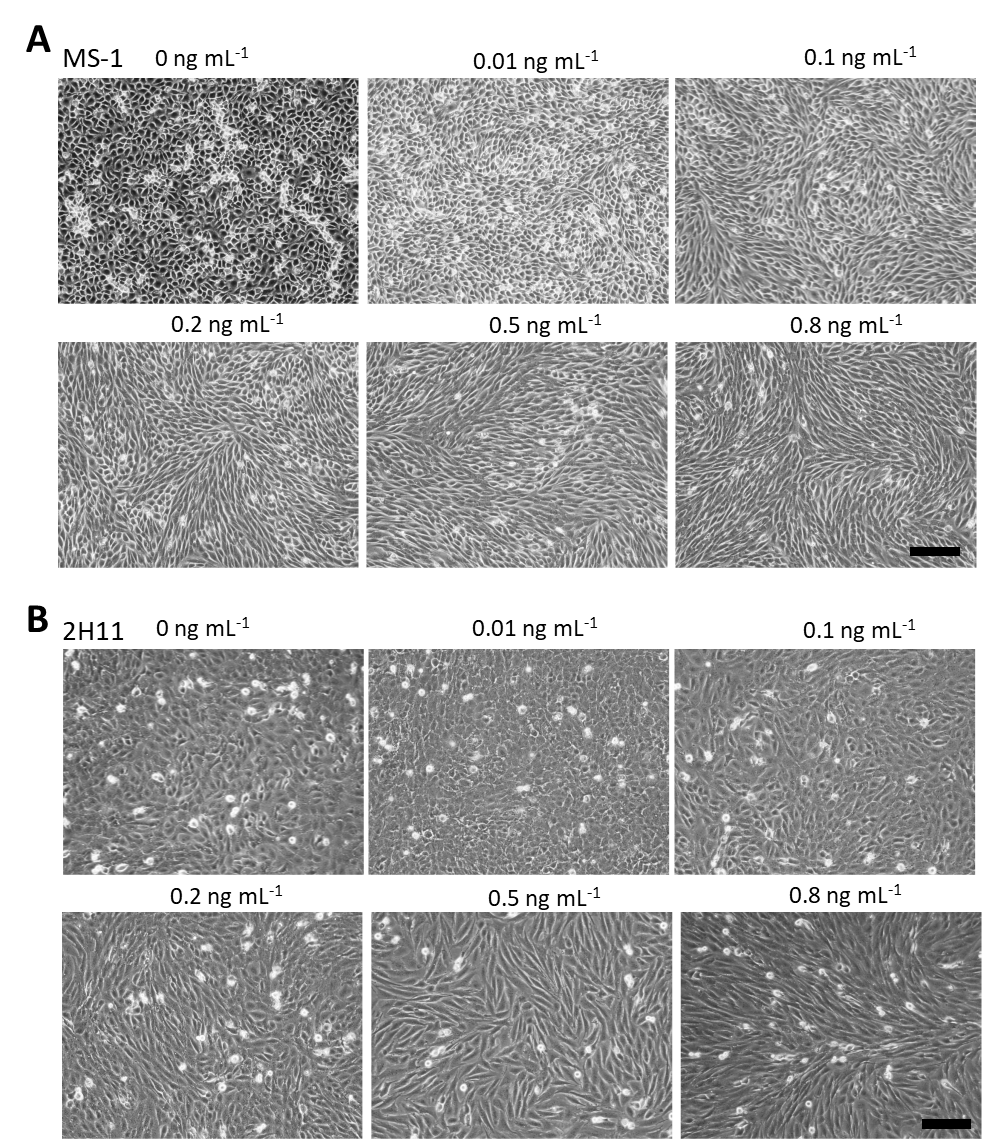


**Figure S13** Dose-dependent TGF-β2-induced EndMT-related cell morphological changes in MS-1 and 2H11 cells. **(A)** Assessing MS-1 cell morphological changes induced by TGF-β2 at difference concentration. Brightfield microscopy image analysis of MS-1 cells in the absence or presence of TGF-β2 (0-0.8 ng mL^-1^) treatment for 3 days showed distinct cell morphologies, i.e. cobblestone or fibroblast-like, respectively. Scale bar: 200 μm. **(B)** Assessing 2H11 cell morphological changes induced by TGF-β2 at difference concentrations. Brightfield microscopy image analysis of 2H11 cells in the absence or presence of TGF-β2 (0-0.8 ng mL^-1^) for 3 days showed distinct cell morphologies, i.e. cobblestone or fibroblast-like, respectively. All the experiments were repeated three times. Scale bar: 200 μm.


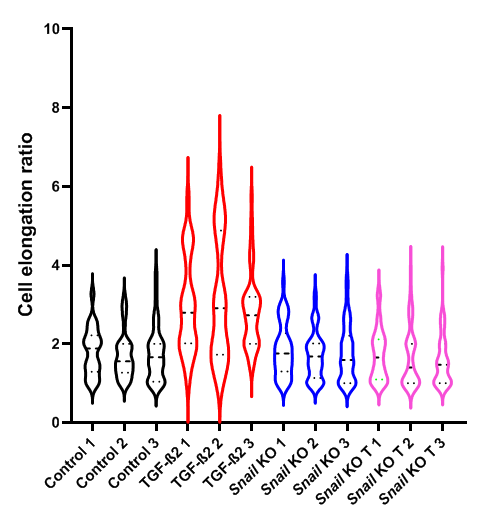


**Figure S14** Depletion of *Snail* attenuates TGF-β2-induced morphological changes in MS-1 cells. Assessment of cell morphological changes shown in Figure 3D measured as cell elongation ratio induced by TGF-β2 (0.1 ng ml^-1^) or vehicle control for 3 days in parental and *Snail* KO MS-1 cells. Results from three independent experiments are shown (labelled with 1, 2 and 3).


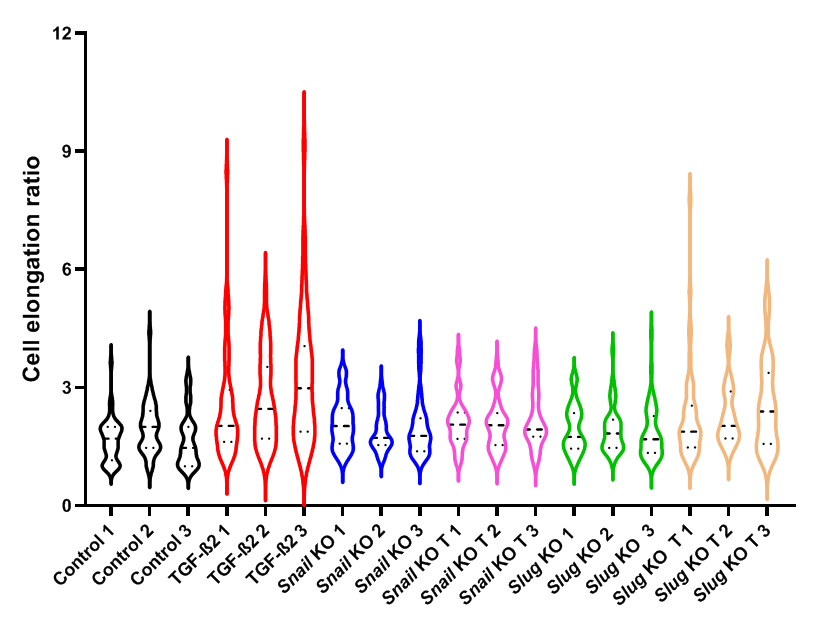


**Figure S15** Depletion of *Snail* or *Slug* attenuates TGF-β2-induced morphological changes in 2H11 cells. Assessment of cell morphological changes shown in Figure 4C measured as cell elongation ratio induced by TGF-β2 (0.2 ng ml^-1^) or vehicle control in parental, *Snail* KO and *Slug* KO 2H11 cells. Results from three independent experiments are shown (labelled with 1, 2 and 3).


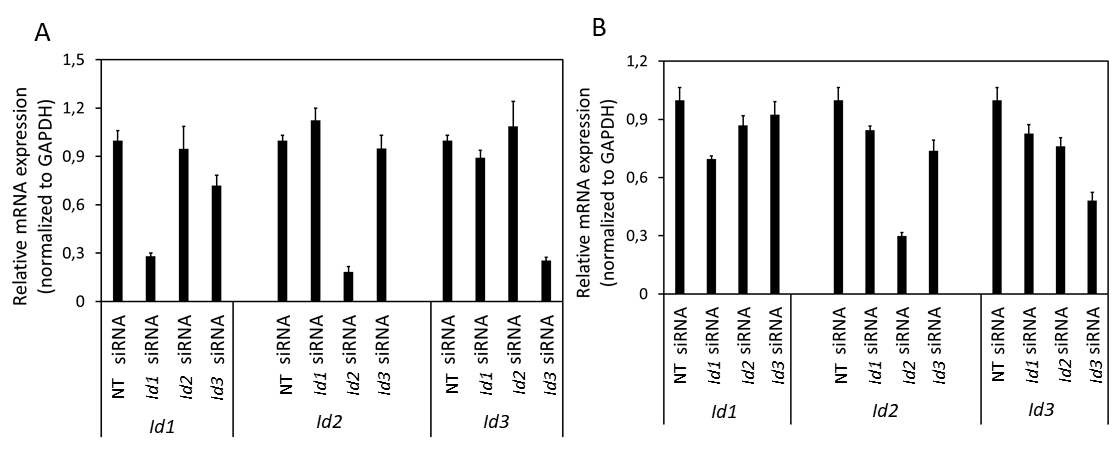


**Figure S16** Effects of siRNA-mediated knockdown of one *Id* gene on expression of two other *Id* genes. **A** RT-qPCR analysis of *Id1*, *Id2* and *Id3* mRNA expression in siRNA-mediated non-targeting (NT), *Id1*, *Id2* or *Id3* suppressed MS-1 cells. **B** RT-qPCR analysis of *Id1*, *Id2* and *Id3* mRNA expression in siRNA-mediated *Id1*, *Id2* or *Id3* suppressed MS-1 cells. The experiments were repeated three times and representative results are shown.


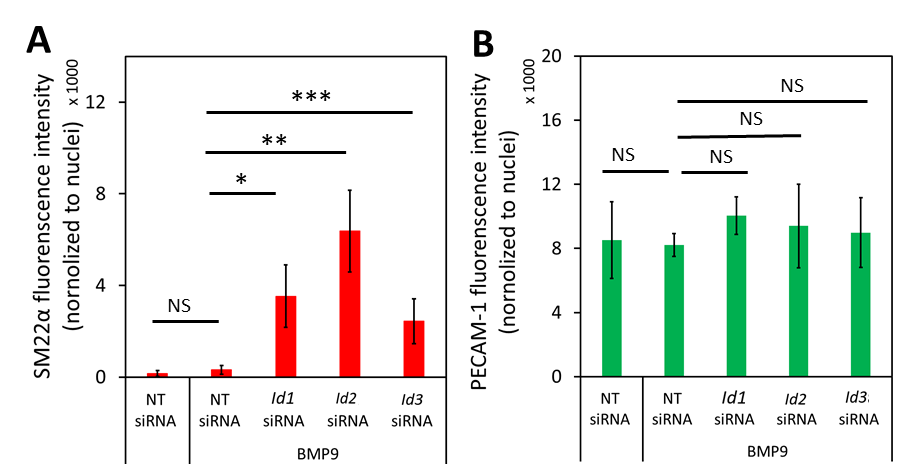


**Figure S17** BMP9 induces EndMT in *Id1/2/3* knockdown MS-1 cells. **(A-B)** Mean fluorescence intensity of SM22α (**A**) and PECAM-1 (**B**) from the non-targeting (NT) knockdown, *Id1* knockdown, *Id2* knockdown and *Id3* knockdown MS-1 cells after incubation in medium containing BMP9 (5 ng ml^-1^) or medium containing ligand buffer (vehicle control) for 3 days. At least six representative images from three independent experiments were quantified. Results are expressed as mean ± SD. NS, not significant; **p* < 0.05, ***p* < 0.005, ****p* < 0.001.


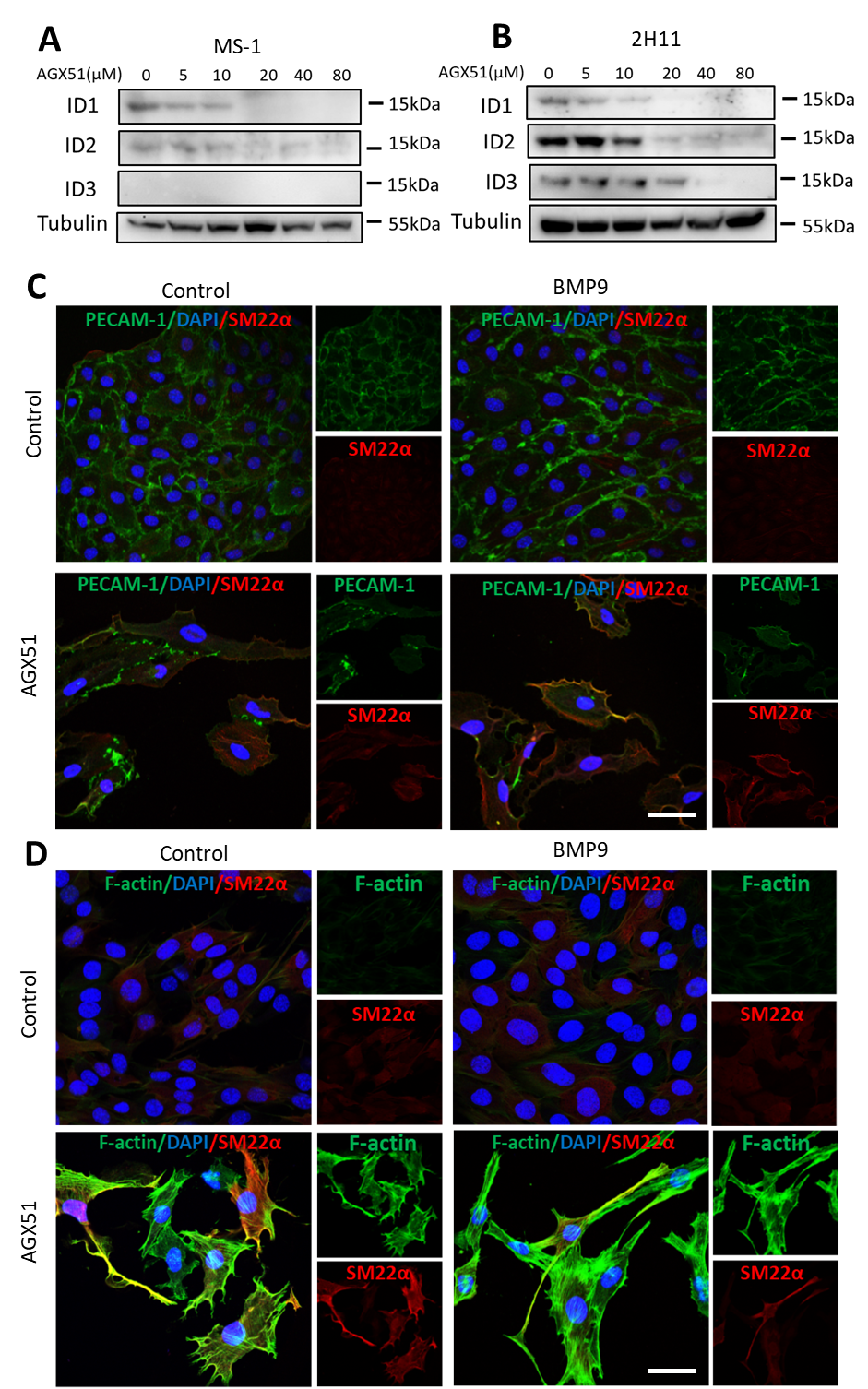


**Figure S18** Id proteins inhibitor AGX51 triggers EndMT. **(A-B)** Western blot analysis of AGX51 regulated the expression of ID1, ID2 and ID3 proteins in MS-1 cells (**A**) and 2H11 cells (**B**) at different concentration (0-80 μM) after 24 h. (**C)** Fluorescence microscopy analysis of endothelial and mesenchymal markers in MS-1 cells. MS-1 cells were incubated in medium containing BMP9 (5 ng mL^-1^) or AGX51 (20 µM) or both BMP9 (5 ng mL^-1^) and AGX51 (20 µM). AGX51 was added 24 h before stimulating with BMP9 for 2 more days. Expression of endothelial cell marker PECAM-1 (green) and mesenchymal cell marker SM22α (red) in nuclei (blue) stained MS-1 cells were assessed by using immunofluorescent staining. Scale bar: 50 μm. (**D)** Fluorescence microscopy analysis of endothelial and mesenchymal markers in 2H11 cells. 2H11 cells were incubated in medium containing BMP9 (5 ng mL^-1^) or AGX51 (20 µM) or both BMP9 (5 ng mL^-1^) and AGX51 (20 µM). AGX51 was added 24 h before stimulating with BMP9 for 2 more days. Expression of mesenchymal cell markers F-actin (green) and SM22α (red) in nuclei (blue) stained 2H11 cells were assessed by using immunofluorescent staining. The experiments were repeated three times and representative results are shown. Scale bar: 50 μm.


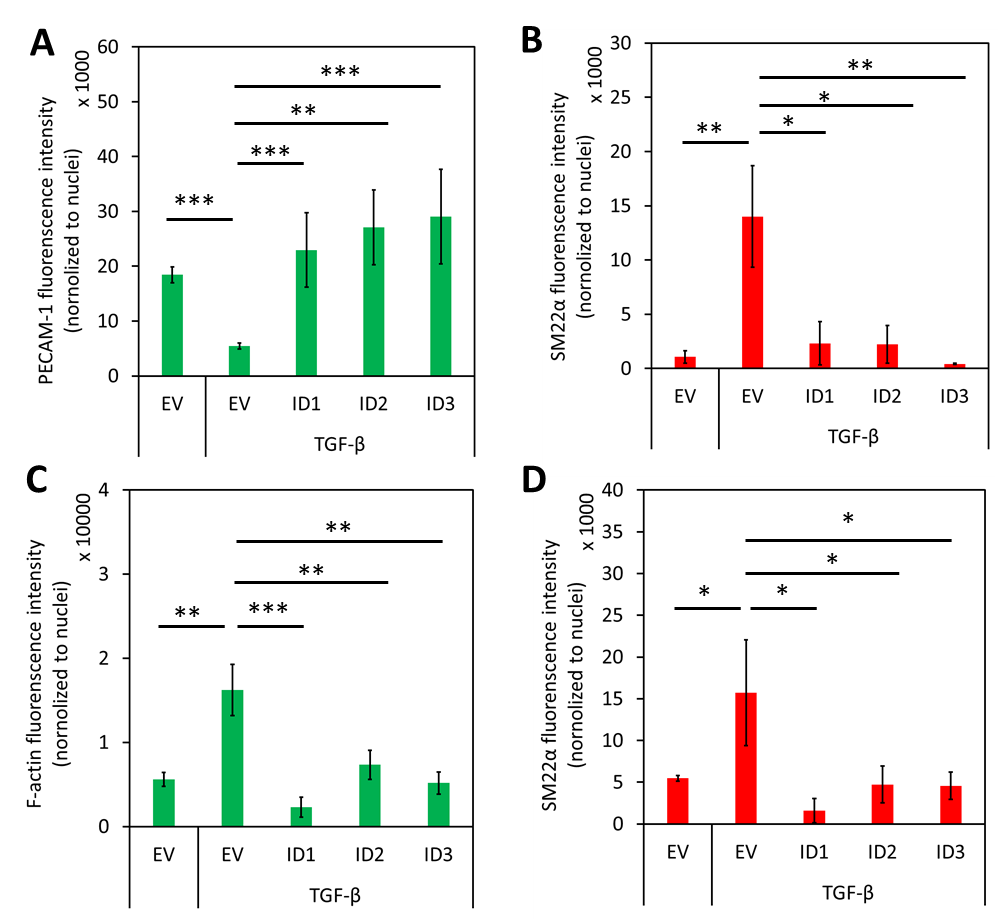


**Figure S19** ID proteins antagonize TGF-β2-induced EndMT. **(A-B)** Mean fluorescence intensity of PECAM-1 (**A**) and SM22α (**B**) from empty vector (EV) expressing MS-1, ID1-overexpressing, ID2-overexpressing and ID3-overexpressing MS-1 cells that were incubated in medium containing TGF-β2 (1 ng ml^-1^) or medium containing ligand buffer (control) for 3 days. At least six representative images from three independent experiments were quantified. Results are expressed as mean ± SD. ***p* < 0.005, ****p* < 0.001. **(C-D)** Mean fluorescence intensity of F-actin (**C**) and SM22α (**D**) from empty vector (EV) expressing 2H11, ID1-overexpressing, ID2-overexpressing and ID3-overexpressing 2H11 cells that were incubated in medium containing TGF-β2 (1 ng ml^-1^) or medium containing ligand buffer (vehicle control) for 3 days. At least six representative images from three independent experiments were quantified. Results are expressed as mean ± SD. ***p* < 0.005, ****p* < 0.001.


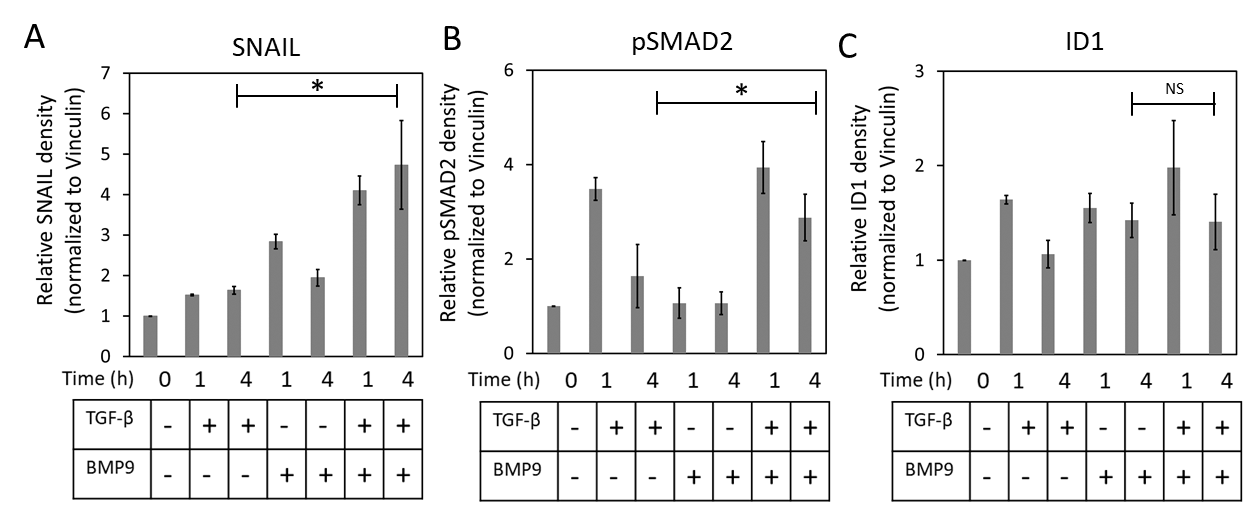


**Figure S20** BMP9 enhances TGF-β2 induced pSMAD2 and SNAIL expression. Quantification of Western blot results shown in Figure 8D of SNAIL (**A**), pSMAD2 (**B**) and ID1 (**C**) expression levels upon stimulation for 1 h and 4 h of MS-1 cells with vehicle control, TGF-β2 (1 ng ml^-1^) and/or BMP9 (5 ng ml^-1^). Values were normalized using Vinculin as a loading control. Results from at least three independent experiments were integrated. Results are expressed as mean ± SD. NS, not significant; **p* < 0.05.

**Figure S21** Uncropped immunoblot images used in the figures. The Figure numbers refer to the (main) Figure to which the images correspond.

**Figure 2B**

**
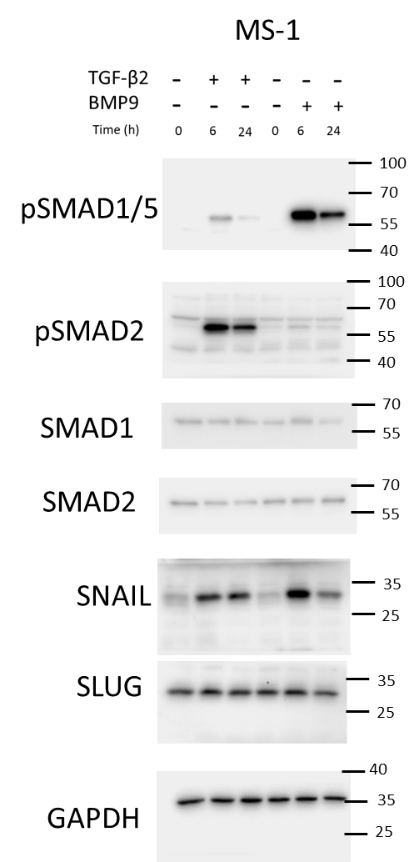
**

**Figure 2G**

**
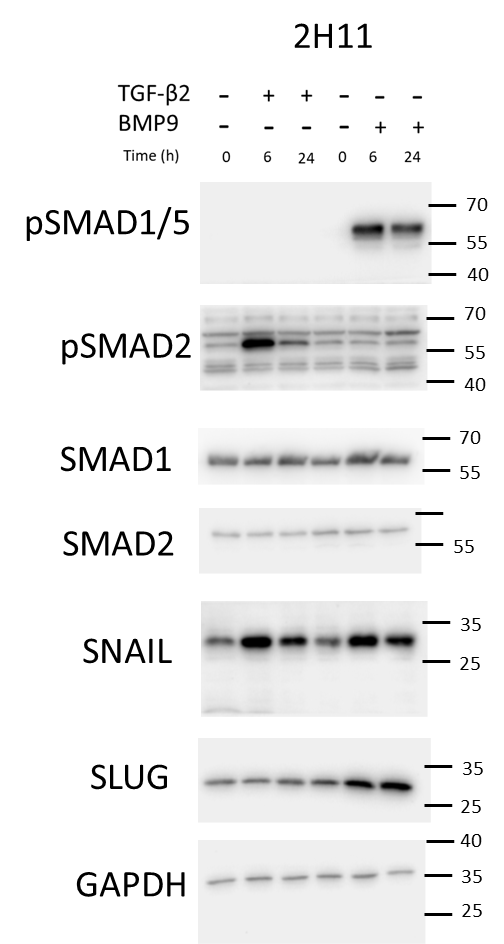
**

**Figure 2E**

**
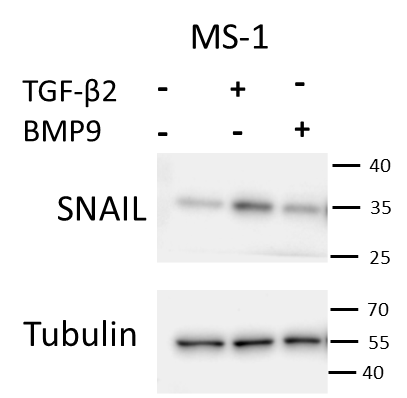
**

**Figure 2J**

**
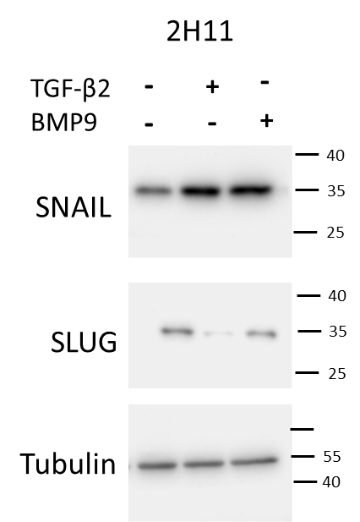
**

**Figure 3A**

**
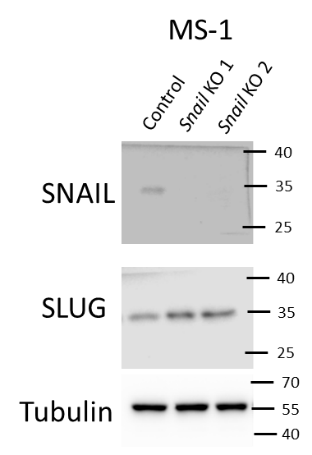
**

**Figure 4A**

**
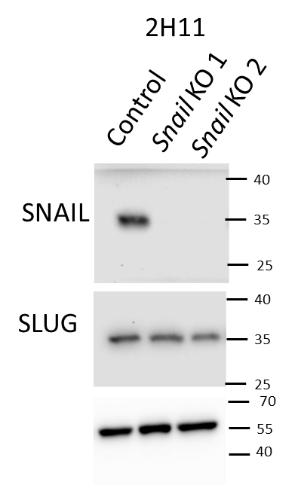
**

**Figure 4B**

**
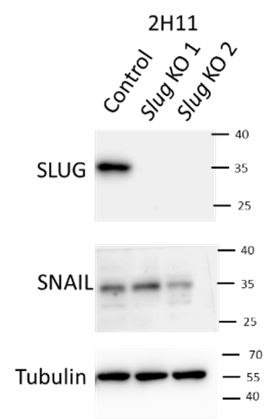
**

**Figure 7D**

**
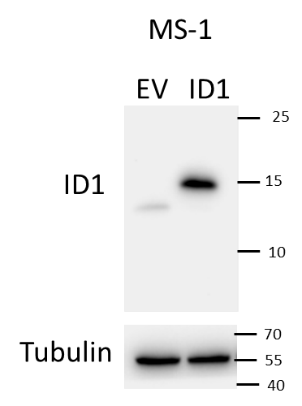
**

**Figure 7E**

**
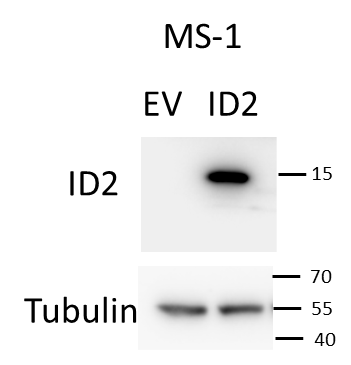
**

**Figure 7F**

**
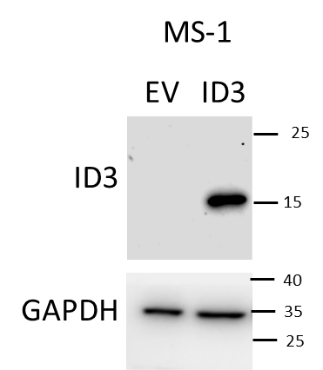
**

**Figure 7J**

**
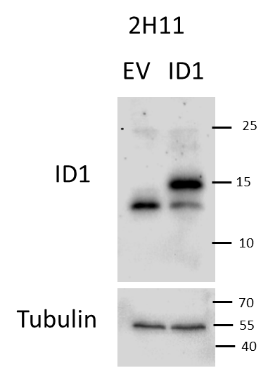
**

**Figure 7K**

**
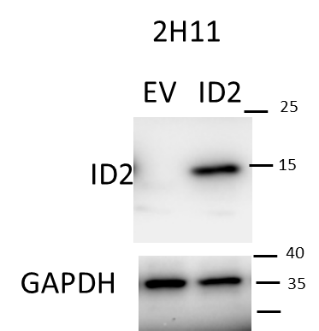
**

**Figure 7L**

**
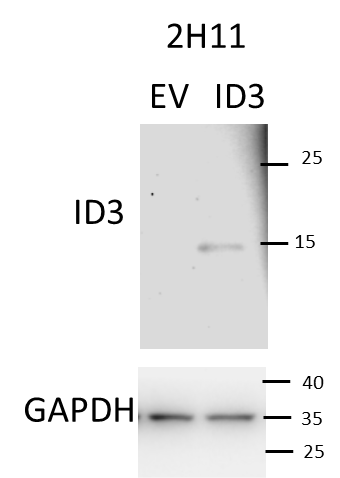
**

**Figure S7B**

**
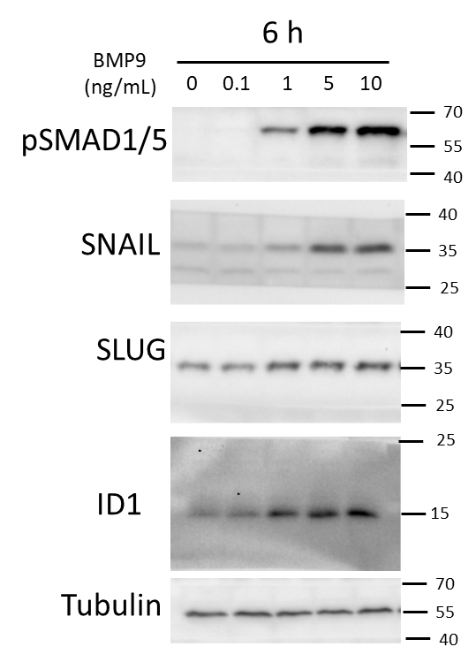
**

**Figure S7A**

**
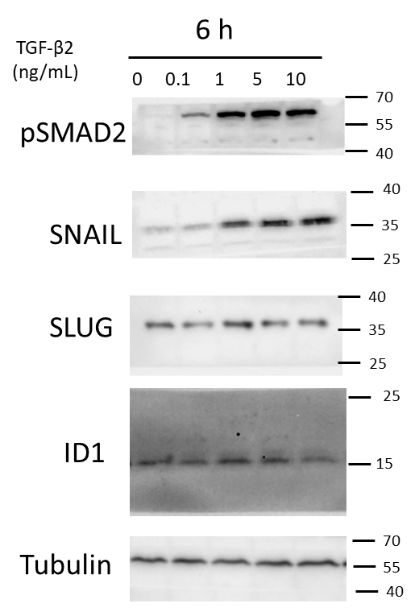
**

**Figure 8D**

**
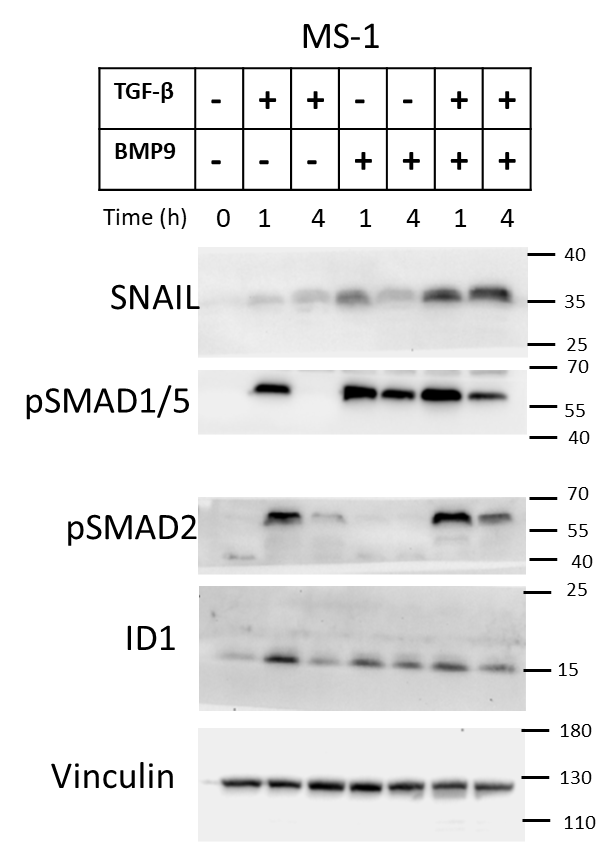
**

**Figure S11**

**
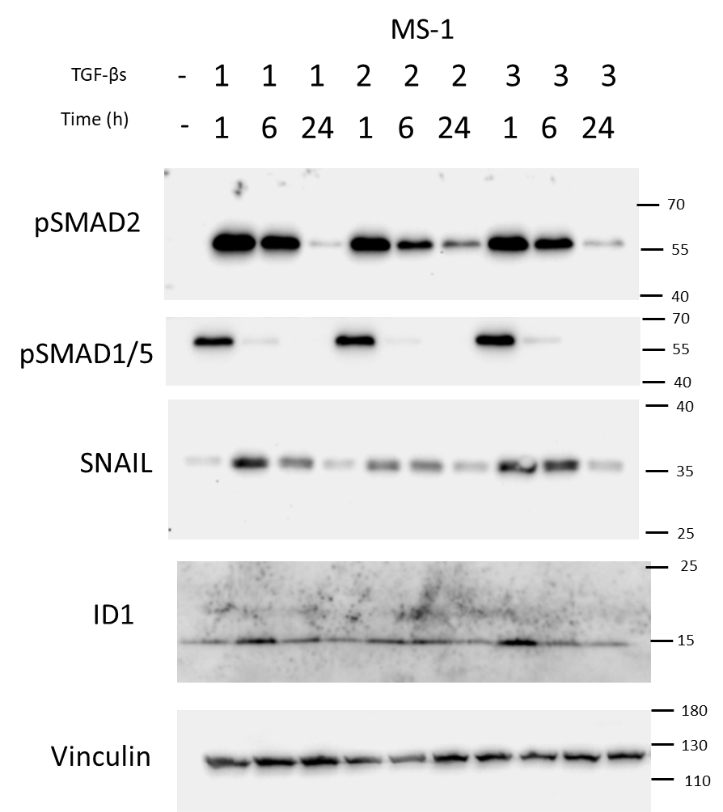
**

**Figure S10A**

**
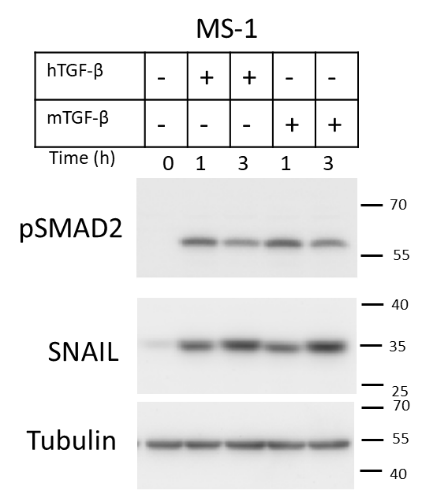
**

**Figure S10B**

**
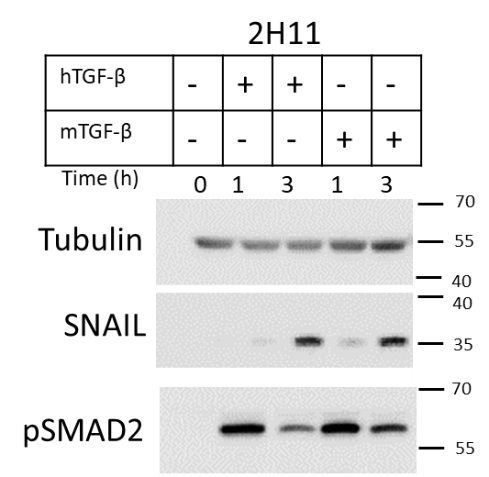
**

**Figure S10C**

**
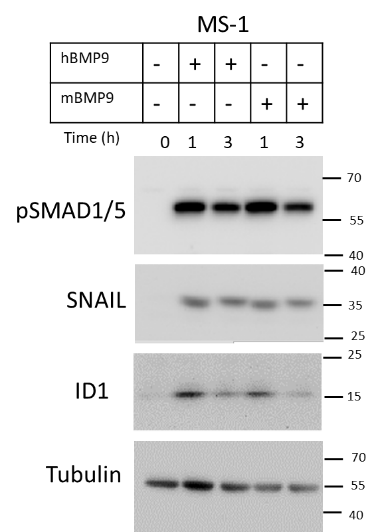
**

**Figure S10D**

**
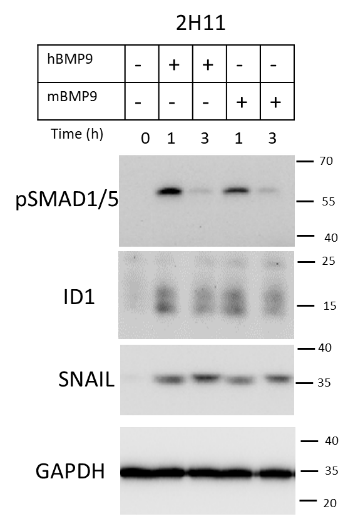
**

**Figure S18A**

**
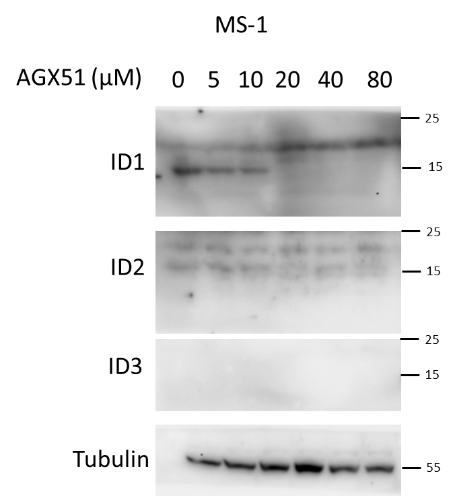
**

**Figure S18B**

**
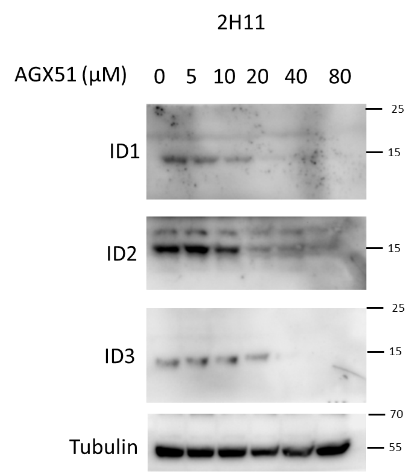
**

**Figure S22 Replicate examples**

**Figure 2B**

**
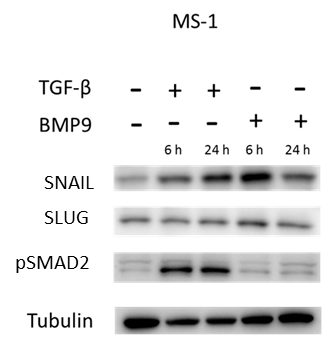
**

**Figure 2G**

**
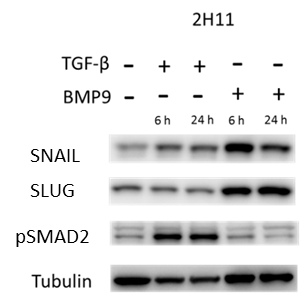
**

**Figure 2E**

**
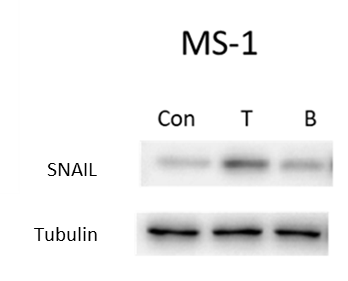
**

**Figure 2J**

**
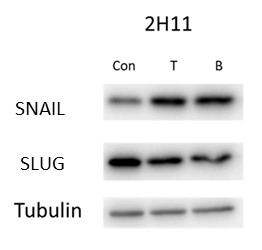
**

**Figure 8D**

**
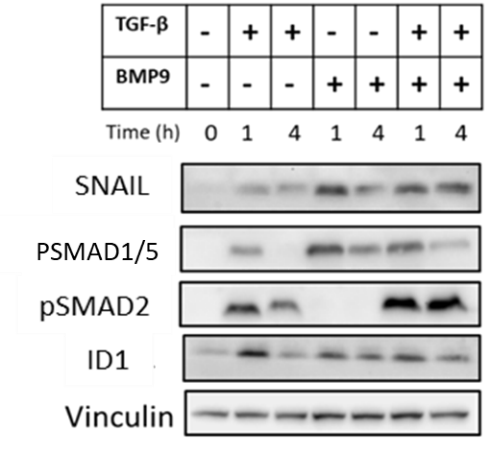
**
